# An Enlarged Profile of Uremic Solutes

**DOI:** 10.1371/journal.pone.0135657

**Published:** 2015-08-28

**Authors:** Hisae Tanaka, Tammy L. Sirich, Natalie S. Plummer, Daniel S. Weaver, Timothy W. Meyer

**Affiliations:** 1 The Departments of Medicine, VA Palo Alto HCS and Stanford University, Palo Alto, CA, United States of America; 2 Bioinformatics Research Group, SRI International, Menlo Park, CA, United States of America; Medical University of Graz, AUSTRIA

## Abstract

Better knowledge of the uremic solutes that accumulate when the kidneys fail could lead to improved renal replacement therapy. This study employed the largest widely available metabolomic platform to identify such solutes. Plasma and plasma ultrafiltrate from 6 maintenance hemodialysis (HD) patients and 6 normal controls were first compared using a platform combining gas and liquid chromatography with mass spectrometry. Further studies compared plasma from 6 HD patients who had undergone total colectomy and 9 with intact colons. We identified 120 solutes as uremic including 48 that had not been previously reported to accumulate in renal failure. Combination of the 48 newly identified solutes with those identified in previous reports yielded an extended list of more than 270 uremic solutes. Among the solutes identified as uremic in the current study, 9 were shown to be colon-derived, including 6 not previously identified as such. Literature search revealed that many uremic phenyl and indole solutes, including most of those shown to be colon-derived, come from plant foods. Some of these compounds can be absorbed directly from plant foods and others are produced by colon microbial metabolism of plant polyphenols that escape digestion in the small intestine. A limitation of the metabolomic method was that it underestimated the elevation in concentration of uremic solutes which were measured using more quantitative assays.

## Introduction

Numerous solutes accumulate in the plasma when the kidneys fail[1]. Such solutes were initially identified individually using the methods of classic organic chemistry. Recent technical developments have greatly accelerated the process of uremic solute identification[2]. "Metabolomic" methods combining mass spectrometry with chromatography have allowed investigators to simultaneously estimate the levels of large numbers of solutes in single plasma samples[3–9]. The current study applied the largest widely available metabolomic platform to identify previously unrecognized uremic solutes. A careful effort was made to combine our results with those of other studies and thereby provide an extended list of known uremic solutes. Because efforts to limit solute production could in the future be combined with renal replacement therapy to lower solute levels, we paid further attention to the source of solutes. Comparison of samples from hemodialysis patients with and without colons identified new solutes made by colon microbes. Discussion of colon-derived solutes has recently been focused on compounds produced by microbial metabolism of amino acids[10]. Our results suggest that a larger number of colon-derived solutes are produced by microbial metabolism of plant polyphenols which escape digestion in the small intestine. Knowledge of the plant sources and microbial metabolic pathways giving rise to these solutes could eventually guide efforts to suppress their production.

## Materials and Methods

Pre-treatment plasma samples obtained by Sirich et al.[11] from six hemodialysis patients and six control subjects were subjected to metabolomic analysis. Characteristics of the subjects are summarized in S1 Table. Plasma ultrafiltrate obtained using Nanosep 30 separators was concentrated four-fold by drying and resuspension in water. Samples were shipped to Metabolon, Inc. for metabolomic profiling. The methods used by Metabolon for identification and estimation of the relative amounts of individual compounds in biologic samples have been described in detail [12, 13]. In brief, all samples are deproteinized with methanol and then aliquoted and dried so that in this study plasma and 4-fold concentrated plasma ultrafiltrate were prepared in the same manner. Individual aliquots are differently reconstituted for analysis by GC-MS and to LC-MS in both positive and negative modes. Samples are run with retention time markers to allow correction for variability in retention times over multiple runs. Mass spectrograms are then compared with those in a library which has been prepared by analyzing more than 2400 purified compounds using the same methods. A single "quant-ion" is selected from either the GC/MS, LC/MS positive, or LC/MS negative data which is considered to best represent each individual compound in the sample set as a whole. Software rates each compound's identification in individual samples based on retention time and comparison of the ion profiles with those recorded in the library. The peak area for the quant-ion is automatically recorded as representing the compound when the quality of identification is considered high and hand-checked when the quality of identification is considered intermediate. No value is recorded for samples in which identification does not meet a threshold value. Because internal standards cannot be employed when so many compounds are assayed, peak areas provide only an estimate or relative concentration.

Metabolomic analysis using these methods identified a total of 459 solutes present in at least some samples from patients and/or normal subjects. To identify uremic solutes, analysis was restricted to a subset of 397 of these solutes detected in the plasma or plasma ultrafiltrate from at least five of the six hemodialysis patients. Solutes were then classified as uremic by two criteria. Solutes were first considered uremic if they were present in plasma or plasma ultrafiltrate from at least five of six patients but detected in none or only one of the normal controls. When solutes were detected in plasma or plasma ultrafiltrate from at least two controls, they were considered uremic if the average peak area in patients exceeded the average peak area in controls by at least 2.5 fold and if the value for *q*, the false discovery rate, was less than 0.05. Values for *q* were calculated using Q-VALUE (http://genomics.princeton.edu/storeylab/qvalue/) from *p* values calculated using Welch's test after log transformation of the mass spectrometric peak areas. A total of 126 solutes met one of the two criteria for classification as uremic. Six of these solutes were excluded because the results obtained in the plasma and plasma ultrafiltrate were discordant, leaving a total of 120 solutes classified as uremic as listed in S2 Table. Chemical names used are from the HMDB for solutes found in that database and in other cases are those used by Metabolon, Inc. except that 2-methoxyphenol sulfate replaces the HMDB name O-methylcatechol-O-sulfate.

Additional analysis was performed to identify uremic solutes derived from colon microbes. Plasma samples obtained by Aronov et al.[7] in nine hemodialysis patients who had intact colons and six hemodialysis patients who had total colectomies were re-analyzed on a newer Metabolon platform using a method incorporating an orbitrap mass spectrometer for LC-MS. Ultrafiltrate was not analyzed because of the limited plasma volume available. A total of 581 compounds were detected in the plasma of at least seven of the nine hemodialysis patients with intact colons. Solutes were classified as colon-derived if the concentration in patients with colons was greater than in those without colons with a false discovery rate *q* < 0.05 calculated as described above. Microbial strains with metabolic pathways containing precursors of colon-derived uremic solutes were identified by querying the BioCyc, HMDB, and Human Microbiome Project databases[14–16]. As listed in S3 Table, 12 of the 120 solutes categorized as uremic in our initial analysis were not detected in the samples analyzed using the newer platform, and the question whether these solutes were colon-derived was thus not addressed.

Levels of selected solutes were also measured using more quantitative assays incorporating reagent standards in known concentrations. Urea nitrogen and creatinine in plasma were measured by the clinical laboratory and hippurate, phenylacetylglutamine, indoxyl sulfate, and p-cresol sulfate in plasma and plasma ultrafiltrate were measured by stable isotope dilution LC/MS/MS as previously described[11].

All clinical studies were conducted according to the Declaration of Helsinki and approved by the IRB of Stanford University: written consent was obtained from subjects who provided samples.

## Results

Metabolomic profiling identified 397 named solutes in pre-treatment plasma samples from at least five of six maintenance hemodialysis patients. A total of 120 of these solutes were identified as uremic by comparison of samples from patients and from normal controls, as listed in S2 Table. A literature search was conducted to determine if solutes found in the current study had been previously identified as uremic. A list was first compiled of solutes listed in reviews and in studies identifying multiple uremic solutes by mass spectrometry[1, 2, 4–9, 17–19]. Solutes identified as uremic in the current study but not listed in these reports were then searched individually in PubMed and the EUTox database. These searches identified an additional 28 solutes previously reported as uremic. No previous reports were found for 48 of the 120 solutes identified as uremic in the present study, and these were therefore considered novel uremic solutes. A combined list of 278 uremic solutes, comprising 230 we found in previous reports and 48 newly identified in the present study, along with references, values for exact mass, and note of inclusion in the Human Metabolome Database (HMDB: http://www.hmdb.ca/)[15] is provided in S4 Table. This combined list of uremic solute includes some solutes which were detected in plasma in the current study but not categorized as uremic by the criteria we employed. These solutes are listed separately in S5 Table.

Many of the 120 solutes identified as uremic in the current study belonged to groups with common characteristics. The largest single group comprised 30 modified amino acids and amino acid degradation products, as listed in Table 1. Nine of these solutes were N-acetylated forms of proteinogenic amino acids, of which only two had previously been identified as uremic solutes. There were 13 other modified amino acids of which 8 were newly identified as uremic solutes. These included the N-acetylated forms of 1- and 3-methylhistidine and of the plant amino acid alliin and N2,N5-diacetylornithine. Also included in Table 1 are 8 compounds known to be produced by amino acid degradation in mammalian cells, of which 2 were newly identified as uremic solutes.

**Table 1:** Modified Amino Acids and Amino Acid Degradation Products.

Solute	Mass	HD/Nl		Previously Identified as Uremic
		Plasma		
	Da	Total	Ultrafiltrate	
**N-Acetylated Standard Amino Acids**				
N-Acetyl-L-alanine	131	6	4	x
N-Acetylserine	147	14	2	
N-Acetylproline	157			
N-Acetylvaline	159	3	2	
N-Acetylthreonine	161	5	4	
N-Acetyl-L-methionine	191	8	6	
N-Acetylhistidine	197	3	5	
N-Acetyl-L-phenylalanine	207	3	5	
N-Acetyltryptophan	246			x
**Other Modified Amino Acids**				
Beta-Alanine	89	4	3	
3-Methylhistidine	169	3	3	x
1-Methylhistidine	169	7		x
N-Formyl-L-methionine	177	3	3	
Homocitrulline	189	5	7	x
N-acetyl-3-methylhistidine*	211	10	14	
N-acetyl-1-methylhistidine*	211	7	4	
N2,N5-diacetylornithine	216	4	4	
N-Acetyl alliin* ^,^ ^†^	219	13	8	
Prolylhydroxyproline	228	9	10	x
L-gamma-glutamyl-L-isoleucine	260	3	4	
O-sulfo-L-tyrosine	261	11	11	
C-mannosyltryptophan	366	16	13	x
**Amino Acid Degradation Products**				
Imidazolepropionic acid	140		2	
Tiglylglycine	157			x
Isovalerylglycine	159		12	x
Quinolinic acid	167	10	6	x
Indoleacetic acid	175	4	12	x
Kynurenic acid	189	7		x
Indolelactic acid	205	3		x
Indoleacetyl glutamine	303		67	

The HD/Nl concentration ratios in pretreatment samples from dialysis patients and normal controls were calculated only when measureable peak areas were obtained in at least two control samples. References to prior reports are included in S2 Table.

* indicates a solute for which a reagent standard was not run but for which identity was considered well established by MS/MS.

^†^ indicates a solute detected in only 5 of 6 dialysis patients.

A second group comprised 24 phenyl and indole compounds not regularly produced by amino acid degradation in mammalian cells, as listed in Table 2. Many of these compounds were conjugates, with 11 precursor compounds conjugated with sulfate, 5 with glycine, and 1 with glutamine. Sixteen compounds had been previously identified as uremic, while 8 were considered to be novel. One of the novel solutes, homovanillic acid sulfate, was previously described as a uremic solute in the unconjugated form[4, 5].

**Table 2:** Phenyl and Indole Compounds.

Solute	Mass	HD/Nl		Previously Identified as Uremic
		Plasma		
	Da	Total	Ultrafiltrate	
**Phenyl Compounds**				
Phenylacetic acid ^†^	136	4		x
2-Aminobenzoic acid	137	3	7	x
4-Hydroxyphenylacetic acid	152	66	70	x
Vanillic acid	168	6		x
Phenol sulphate	174	8	13	x
Hippuric acid	179	21	32	x
p-Cresol sulfate	188	13	21	x
2-Aminophenol sulphate	189	8	10	
Pyrocatechol sulfate	190	3	3	x
Phenylacetylglycine	193			x
4-Hydroxyhippuric acid	195	66	99	x
3-Hydroxyhippuric acid	195	7	9	x
Vanillylmandelic acid	198	15	24	x
4-Vinylphenol sulfate	200	11		
2-Methoxyphenol sulphate	204	25	41	
3-Methylcatechol sulfate ^‡^	204	5		
4-Methylcatechol sulfate	204	3	8	x
Cinnamoylglycine	205	12		x
3-[3-(Sulfooxy)phenyl]propanoic acid	246	13	44	
Homovanillic acid sulfate	262			
Alpha-N-Phenylacetyl-L- glutamine	264	44	32	x
**Indole Compounds**				
Indole-3-methyl acetate	189			
2-Oxindole-3-acetate	191			
Indoxyl sulfate	213	7	20	x

The HD/Nl concentration ratios were calculated only when measureable peak areas were obtained in at least two control samples. References to prior reports are included in S2 Table.

^‡^ indicates that the analytic method did not distinguish which OH group on 3-Methylcatehol had been sulfated.

^†^ indicates a solute detected in only 5 of 6 dialysis patients.

A further group of uremic solutes included compounds potentially derived from pharmaceuticals, as listed in Table 3. These compounds included pantothenic acid and riboflavin which the patients were receiving in a vitamin supplement and pyridoxic acid, a metabolite of pyridoxine contained in the supplement. Other compounds potentially derived from pharmaceuticals included the aspirin metabolite salicyluric acid and its glucuronide and the acetaminophen metabolite 2-hydroxyacetaminophen sulfate.

**Table 3:** Solutes Potentially Derived From Medications.

Solute	Mass	HD/Nl		Previously Identified as Uremic
		Plasma		
	Da	Total	Ultrafiltrate	
Saccharin	183	22		
4-Pyridoxic acid	183	74	93	x
Salicyluric acid	195	49		x
Pantothenic acid	219	8	7	x
2-Hydroxyacetaminophen sulfate* ^,^ ^†^	247			
Salicyluric glucuronide*	371	315		x
Riboflavin^†^	376		12	x

The HD/Nl concentration ratios were calculated only when measureable peak areas were obtained in at least two control samples. References to prior reports are included in S2 Table.

* indicates a solute for which a reagent standard was not run but for which identity was considered well established by MS/MS.

^†^ indicates a solute detected in only 5 of 6 dialysis patients.

The remaining compounds identified as uremic are listed in Table 4, including 38 compounds previously identified as uremic and 21 compounds considered novel. Small groups of compounds with related properties appear at the top of the list. There were 8 polyols, all but one of which had previously been identified as uremic solutes[20, 21]. There were also 4 methyl urates which can be derived from caffeine and other methylxanthines, 4 acyl carnitines, 4 modified nucleosides, and 3 vitamin E metabolites. Additional compounds are listed in order of increasing molecular mass.

**Table 4:** Other Uremic Solutes.

Solute	Mass	HD/Nl		Previously Identified as Uremic
		Plasma		
	Da	Total	Ultrafiltrate	
**Polyols**				
D-Threitol	122	18	5	x
Erythritol	122	4	3	x
Arabitol ^§^	152	10	9	x
Levoinositol	180	16	24	x
Myoinositol	180	11	6	x
Scyllitol	180	5	3	x
Galactitol	182			
Mannitol	182	49	36	x
**Methyl Urates**				
7-Methyluric acid^†^	182	40	49	
1-Methyluric acid	182	12	11	x
1,7-Dimethyluric acid	196	4	5	x
1,3,7-Trimethyluric acid	210	4	3	x
**Acyl-Carnitines**				
Isobutyryl-L-carnitine	231	5	4	
Phenylcarnitine*	237			
Glutarylcarnitine	275	5		x
3-Methylglutarylcarnitine	289	37	31	x
**Modified Nucleosides**				
Pseudouridine	244	7	5	x
5'-Methylthioadenosine	297	3	4	
N2,N2-Dimethylguanosine	311	12	11	x
N6-Carbamoyl-L-threonyladenosine	412	24	20	x
**Vitamin E Metabolites**				
Gamma-CEHC	248	4		x
Gamma-CEHC glucuronide*	424			
Alpha-CEHC glucuronide*	454			
**Other**				
Urea	60	3	2	x
Glycine	75	3	2	x
3-Aminoisobutanoic acid	103	2	3	x
Creatinine	113	7	6	x
Fumaric acid	116	4	4	x
Erythronic acid	136	21	18	x
Threonic acid	136	4	1	x
Methylimidazoleacetic acid	140	12	9	
Proline betaine	143	3	3	x
Isobutyrylglycine	145		6	
4-Acetamidobutanoic acid	145	19	26	x
4-Guanidinobutanoic acid	145	3	5	x
Tartaric acid	150		38	
D-Xylose	150	16	7	
L-Arabinose	150	5	5	
N1-Methyl-2-pyridone-5-carboxamide	152	5	5	x
2,5-Furandicarboxylic acid	156			
Orotic acid	156	5	8	x
Allantoin	158	15	12	x
Levoglucosan	162	44	44	
L-Fucose ^†^	164	3	5	
Arabinonic acid	166	16	6	x
L-Xylonate	166	12	26	
2-Furoylglycine	169			x
Citrulline	175	3	2	x
L-Gulonolactone	178	22	25	
Acisoga	184	3	3	
Gluconic acid	196	21	18	x
Pyroglutamylvaline	228			
Cytidine	243	4	3	x
Acetylcarnosine	268	6	5	
N-Acetylneuraminic acid	309	8	7	x
Sucrose	342		35	x
Androsterone sulfate	370	3		x
S-Adenosylhomocysteine	384			x
6-Sialyl-N-acetyllactosamine	674		30	

The HD/Nl concentration ratios were calculated only when measureable peak areas were obtained in at least two control samples. References to prior reports are included in S2 Table.

* indicates a solute for which a reagent standard was not run but for which identity was considered well established by MS/MS.

^§^ indicates that the analysis does not distinguish between the D- and L- forms of arabitol.

^†^ indicates a solute detected in only 5 of 6 dialysis patients.

To further identify the source of the uremic solutes, we re-analyzed plasma samples from a study comparing hemodialysis patients with and without colons[7]. Nine uremic solutes were shown to be colon-derived, as listed in Table 5. Of note, these compounds were all among the phenyl and indole uremic compounds listed in Table 2. Three of them—p-cresol sulfate, indoxyl sulfate, and phenylacetylglutamine—had previously been identified as colon-derived[22]. Query of the BioCyc database identified potential microbial sources for the colon-derived solutes, as further summarized in Table 5.

**Table 5:** Colon-Derived Solutes.

Uremic Solute	Colectomy /Intact	Presumed Microbial Product	Possible Precursors	MetaCyc Frame ID of Presumed Microbial Product	All Microbes	Gut Microbes
Phenylacetic acid	0.03	Phenylacetic acid	Phenylalanine	PHENYLACETATE	3115	154
p-Cresol sulfate ^‡^	0.03	p-Cresol	Tyrosine	CPD-108	4764	449
2-Aminophenol sulphate	0.22	2-Aminophenol	3-Hydroxyanthranilate, dietary aromatic compounds	2-AMINOPHENOL	60	0
	0.22	3-Hydroxyanthranilate	Tryptophan, dietary aromatic compounds	3-HYDROXYANTHRANILATE	821	84
3-Hydroxyhippuric acid	0.01	3-Hydroxybenzoic acid	Chorismate, dietary aromatic compounds	3-HYDROXYBENZOATE	96	2
2-Methoxyphenol sulfate	0.13	Guaiacol	Catechol, dietary aromatic compounds	CPD-400	12	0
4-Methylcatechol sulfate	0.09	4-Methylcatechol	Tyrosine, dietary aromatic compounds	4-METHYLCATECHOL	411	8
Alpha-N-phenylacetyl-L-glutamine ^‡^	0.14	Phenylacetic acid	Phenylalanine	PHENYLACETATE	3115	154
Indoxyl sulfate ^‡^	0.03	Indole	Tryptophan	INDOLE	4782	420
3-(3-(Sulfooxy)phenyl) propanoic acid	0.01	3-(3-Hydroxyphenyl) propanoic acid	Caffeine, dietary aromatic compounds	3-HYDROXYPHENYL-PROPIONATE	345	9

^‡^ indicates solutes previously identified as colon-derived in the dialysis patients. 'Colectomy/Intact' provides the estimated ratio of the solute's concentrations in dialysis patients without and with colons. Unconjugated microbial products from which the uremic solutes are presumed to be derived and some of their possible precursors are then listed along with the Frame IDs for the product compounds in the BioCyc collection of pathway/genome databases (http://biocyc.org).[14] ‘All Microbes’ represents the number of microbial organism databases in BioCyc that contain at least one metabolic pathway containing the compound. ‘Gut Microbes’ represents the number of these organism databases associated with the gastrointestinal tract by the Human Microbiome Project.[16] In the case of 2-Aminophenol sulphate, we list as presumed precursor compounds both the unconjugated compound 2-aminophenol and the closely related compound 3-hydroxyanthranilate.

For selected solutes, we were able to compare the concentration ratios obtained by metabolomic analysis with those obtained by quantitative assays of the same samples. This comparison revealed that metabolomic analysis tended to underestimate the degree to which solute concentrations were elevated in hemodialysis patients as compared to control subjects and did not reliably assess the extent of protein binding, as summarized in Table 6.

**Table 6:** Comparison of Solute Concentration Ratios Obtained by Metabolomic Analysis and by Quantitative Assays.

	Metabolomic			Quantitative		
Solute	HD/Nl		Percent	HD/Nl		Percent
	Plasma		Free HD	Plasma		Free HD
	Total	Ultrafiltrate		Total	Ultrafiltrate	
Urea	3	2.5	39	4		
Creatinine	7	6.2	107	11		
Phenylacetylglutamine	44	32	44	126	156	92
Hippurate	21	32	42	42	81	51
Indoxyl sulfate	7	20	8	30	46	7
p-Cresol sulfate	13	21	6	20	51	6

Creatinine and urea nitrogen were not assayed quantitatively in plasma ultrafiltrate.

## Discussion

This study compared plasma from hemodialysis patients and control subjects using the largest metabolomic platform which is widely available. Among 397 named solutes identified in most of the patients, 120 could be classified as uremic. For 48 of these solutes prior reports of accumulation in renal failure were not found.

It should be emphasized that individual solute concentrations cannot be measured accurately when hundreds of solutes are examined at once. Isotopically labeled internal standards which would correct for differences in ionization efficiency are not employed, and comparison of mass spectrometric peak areas provides only an estimate of relative concentrations. The metabolomic method employed here underestimated the concentration in patients relative to controls for solutes which were also measured with quantitative assays. The failure of mass spectrometric peak areas to increase in proportion to solute concentration in the dialysis patients may reflect not only self suppression of ionization by the analyte in question but also suppression of ionization by other uremic solutes which have similar chromatographic retention time.

It should also be emphasized that criteria used to classify solutes as uremic are arbitrary. We applied this classification when the ratio of average peak areas in patients compared to normals was at least equal to the value of 2.5 obtained for urea in plasma ultrafiltrate, as shown in Table 6. Use of a higher cutoff might have prevented inappropriate classification of some solutes as uremic by chance. Use of a lower cutoff, however, would likely have resulted in failure to identify solutes whose concentrations are truly elevated in renal failure, particularly given our finding that the current metabolomic method underestimated the elevation in concentration for those solutes also measured by quantitative assays. We should note that the most widely cited studies in this area have aimed at an inclusive listing of uremic solutes and therefore included all solutes whose reported concentrations in renal failure patients exceed those in normal subjects[1, 17].

Among the solutes classified as uremic here, several groups with common properties could be distinguished. There were a large number of modified amino acids and amino acid degradation products. The largest single group, including 7 compounds not previously reported as uremic, comprised the N-acetyl forms of standard, proteinogenic amino acids as listed in Table 1. These compounds are presumably released during proteolysis of the more than 80 percent of human body proteins that are N-acetylated during synthesis[23, 24]. Normally, glomerular filtration followed by reabsorption and hydrolysis in the proximal tubule cells allows recovery of the native amino acids[24, 25]. The same process may normally allow recovery of methionine from N-formyl-L-methionine which is an initiating residue in mitochondrial protein synthesis.

Several other uremic solutes which are modified amino acids are derived from muscle. 3-methylhistidine and N-acetyl-3-methylhistidine are derived from breakdown of post-translationally modified muscle proteins and normally excreted in the urine[26]. Other modified amino acids are derived from the muscle dipeptide carnosine. N-acetyl-1-methylhistidine is presumably derived, like 1-methylhistidine, from dietary anserine which is formed from carnosine in many animals but not in humans[27–29]. Beta-alanine, in contrast, is formed from carnosine in humans as well as other animals. The sources of N2,N5-diacetylornithine and O-sulfo-L-tyrosine are less clear, but it seems likely that they are derived from breakdown of proteins containing modified amino acids. This is known to be the source of L-gamma-glutamyl-L-isoleucine which is newly identified here as uremic and of homocitrulline, prolylhydroxyproline, and C-mannosyltryptophan.

Another group of uremic solutes includes phenyl and indole compounds which are not produced by amino acid degradation in mammalian cells. The majority of these compounds are conjugates, including 11 sulfates, 5 glycine conjugates, and 1 glutamine conjugate. Recent studies have focused on the potential toxicity of phenyl and indole compounds derived from catabolism of amino acids, particularly indoxyl sulfate, indoxyl acetate, and p-cresol sulfate[10, 30]. A literature search suggested however that most of the compounds listed in Table 2 are derived not from the amino acids but from various substances found in plant foods, as summarized in S6 Table. Some solutes, such as vanillic acid and indole-3-methyl acetate, are themselves found in plants and can presumably be absorbed intact from plant foods[31, 32]. Other solutes may be derived from the action of colon microbes on plant precursor compounds, as further summarized in S6 Table. Homovanillic acid sulfate and vanillylmandelic acid, by contrast, are mammalian degradation products of dopamine and epinephrine/norepinephrine[33].

The microbial origin of 9 phenyl and indole uremic solutes was confirmed by comparison of samples from patients with and without colons. Three of these solutes—p-cresol sulfate, indoxyl sulfate, and phenylacetylglutamine—are well known as microbially-derived uremic solute while the other 6 had not previously been identified as such[22]. With the exception of phenylacetic acid, the microbially-derived solutes are conjugates. We presume that microbes produce precursor compounds which are then conjugated in the liver or colon wall, as is the case with p-cresol sulfate and indoxyl sulfate. Examination of the BioCyc databases confirmed that there are numerous microbial strains with metabolic pathways containing the precursor compounds, as summarized in Table 5. In all but 2 cases such strains have been associated with the gastrointestinal tract by the Human Microbiome Project[16].

Identifying the sources of uremic solutes may prove ultimately to have clinical value, in that it may prove simpler to reduce solute levels by suppressing solute production than by enhancing solute removal. Efforts to limit production of solutes derived from amino acids by limiting protein intake were largely abandoned after Kt/V_urea_ was adopted as an index of dialysis adequacy. The literature, however, provides very limited support for the current recommendation that dialysis patients maintain a higher than average protein intake[34]. And to the extent that solutes derived from amino acids are toxic, a high protein intake may have ill effects.

The current study emphasizes that production of uremic solutes may also depend on the intake of plant foods. This possibility that increasing plant food intake could increase uremic solute production was recognized when far fewer solutes had been identified[35]. It has since, however, been neglected. Our data suggest that plant phenols and polyphenols may be particularly important sources of uremic solutes. Plants contain thousands of these compounds with an extraordinary variety of structures[36]. Some of the simpler compounds can be absorbed directly. Most of the polyphenols, however, escape digestion in the small intestine and are metabolized by colon microbes[37, 38]. Several grams per day of dietary polyphenols are thereby transformed into new substances foreign to mammalian metabolism which are normally excreted by the kidney, often after conjugation in the liver. Consideration of the health effects of plant phenols has so far focused largely on their potential benefit with particular attention to their potential antioxidant and anticarcinogenic activity.[37, 38] [39] Accumulation of metabolites which are normally excreted by the kidney could turn these putative benefits to harm in patients with renal failure. If this proves to be the case, the production of injurious solutes could be reduced by avoiding certain foods, as the content of polyphenols varies widely among plants. The production of microbially-derived solutes could presumably also be reduced by altering the composition of the colon microbiome or by the use of binding agents.[22, 40, 41]

Another group of solutes whose production could be controlled are those derived, at least in part, from pharmaceuticals as listed in Table 3. There are no natural sources of saccharin and acetaminophen, so accumulation of saccharin and 2-hydroxyacetaminophen sulfate in renal failure is presumably due exclusively to intake of the synthetic compounds. Salicyluric acid and salicyluric glucuronide may in contrast be derived from salicylate in plant foods, but this constitutes a small portion of total salicylate production in patients taking aspirin[42]. All the patients were prescribed a supplement containing vitamins B6 (pyridoxal-5-phosphate, as pyridoxine), B5 (pantothenic acid), and B2 (riboflavin). Supplementation of vitamins B5 and B2 is based on theoretical concern for dialytic loss and not on demonstration of deficiency in dialysis patients[43, 44]. Both compounds are widely available in foods and levels in our patients were above those in controls. Supplementation of vitamin B6 is based on the finding of reduced levels in some studies of unsupplemented patients[43, 44]. Supplementation, however, presumably contributes to the prominent accumulation of the B6 metabolite 4-pyridoxic acid, providing an example of the unintended consequences of drug administration in dialysis patients[45].

Limited knowledge of solute toxicity poses the major barrier to the development of new treatments to reduce solute production or increase solute removal[1, 2]. Metabolomic methods are already being employed to search for solutes associated with outcomes, and such efforts will undoubtedly continue[46]. The present study illustrates not only the capacity of metabolomic profiling to identify solutes but also important limitations of this approach. The major limitation, as noted above, is that accurate quantitation cannot be achieved when large numbers of compounds are examined simultaneously and internal standards for each compound are not included in the analysis. Quantitative accuracy may be particularly limited when different sample matrices, such as plasma and plasma ultrafiltrate, are compared. We analyzed both plasma and plasma ultrafiltrate because we hoped to characterize the extent of protein binding for each solute, which has a large effect on a solute's clearance by dialysis treatment[47, 48]. Potential errors in the estimation of concentrations from metabolomic peak areas, as revealed by the apparent protein binding of urea and phenylacetylglutamine in Table 6, dissuaded us from reporting the binding of solutes not measured using quantitative assays.

A second limitation is that we have not shown that solute accumulation is due to reduced renal clearance. Previous studies have shown that some solutes accumulate in patients with renal failure because of reduced hepatic clearance, presumably because solutes normally cleared by the kidney interfere with hepatic uptake and disposal mechanisms[49]. Also, as has been the case with the great majority of solutes previously categorized as uremic, production rates were not measured. We thus cannot exclude that solute accumulation reflects increased solute production in uremic patients. In the case of solutes made from plant phenols, we doubt that intake of precursor plant foods was high in the dialysis patients, but the production of individual solutes could have been increased due to alterations in the colon microbiome. Because we studied a limited number of subjects, some differences classified as uremic could have arisen from differences in diet, age, and other factors unrelated to renal dysfunction.

A further limitation of metabolomic studies is that no single method can profile all the solutes that accumulate in uremia. Analysis by several platforms was necessary to compile the current list of more than 4000 solutes found in human plasma[50]. And the platform used in this study, while the largest that is widely available, does not include many of the known uremic solutes listed in S4 Table. In some cases the method employed may not provide chromatographic separation. In other cases, standards have not been run on the platform. It should be noted that the plasma metabolome in uremia may be particularly difficult to profile because many uremic solutes remain to be chemically identified. Studies using untargeted high-resolution mass spectrometry have revealed the presence of numerous uremic solutes characterized only by molecular mass for which there are no corresponding entries in the current list of human metabolites[3, 6, 19]. Finally, it is important to emphasize that the designation "uremic," implies only that a compound accumulates when the kidneys fail, and not that it has been shown to cause illness. Referring to such compounds as uremic solutes rather than uremic toxins may serve as a reminder of this distinction. A great deal of additional work is thus required to identify uremic solutes as well as to assess their contributions to clinical outcomes.

## Supporting Information

S1 Table Characteristics of Hemodialysis Patients and Normal Subjects. (DOC)

S2 Table Uremic Solutes Found in the Present Study (n = 120). (DOC)

S3 Table Solutes Classified As Uremic On Original Analysis Which Were Not Detected When Samples From Dialysis Patients With and Without Colons Were Compared. (DOC)

S4 Table Uremic Solutes Found in the Literature and Uremic Solutes Found in the Present Study (n = 278). (DOC)

S5 Table Solutes Detected in Plasma and Categorized as Uremic in Previous Studies But Not in the Present Study (n = 28). (DOCX)

S6 Table Plant Sources of Uremic Phenyl and Indole Solutes. (DOC)

## References

[1] Duranton F, Cohen G, De Smet R, Rodriguez M, Jankowski J, Vanholder R, et al Normal and pathologic concentrations of uremic toxins. J Am Soc Nephrol. 2012;23(7):1258–70. 10.1681/ASN.2011121175 22626821 PMC3380651

[2] Niwa T. Update of uremic toxin research by mass spectrometry. Mass Spectrom Rev. 2011;30(3):510–21. 10.1002/mas.20323 21328600

[3] Kikuchi K, Itoh Y, Tateoka R, Ezawa A, Murakami K, Niwa T. Metabolomic analysis of uremic toxins by liquid chromatography/electrospray ionization-tandem mass spectrometry. J Chromatogr B Analyt Technol Biomed Life Sci. 2010;878(20):1662–8. 10.1016/j.jchromb.2009.11.040 20036201

[4] Rhee EP, Souza A, Farrell L, Pollak MR, Lewis GD, Steele DJ, et al Metabolite profiling identifies markers of uremia. J Am Soc Nephrol. 2010;21(6):1041–51. doi: ASN.2009111132 [pii] 10.1681/ASN.2009111132 20378825 PMC2900954

[5] Toyohara T, Akiyama Y, Suzuki T, Takeuchi Y, Mishima E, Tanemoto M, et al Metabolomic profiling of uremic solutes in CKD patients. Hypertens Res. 2010;33(9):944–52. 10.1038/hr.2010.113 20613759

[6] Sato E, Kohno M, Yamamoto M, Fujisawa T, Fujiwara K, Tanaka N. Metabolomic analysis of human plasma from haemodialysis patients. Eur J Clin Invest. 2011;41(3):241–55. 10.1111/j.1365-2362.2010.02398.x 20955218

[7] Aronov PA, Luo FJ, Plummer NS, Quan Z, Holmes S, Hostetter TH, et al Colonic contribution to uremic solutes. J Am Soc Nephrol. 2011;22(9):1769–76. 10.1681/ASN.2010121220 21784895 PMC3171947

[8] Yu B, Zheng Y, Nettleton JA, Alexander D, Coresh J, Boerwinkle E. Serum metabolomic profiling and incident CKD among African Americans. Clin J Am Soc Nephrol. 2014;9(8):1410–7. 10.2215/CJN.11971113 25011442 PMC4123405

[9] Boelaert J, d'kindt R, Schepers E, Jorge L, Glorieux G, Neirynck N, et al State-of-the-are non-targeted metabolomics in the study of chronic kidney disease. Metabolomics. 2014;10:425–42. 10.1007/s11306-013-0592-z

[10] Vanholder R, Schepers E, Pletinck A, Nagler EV, Glorieux G. The uremic toxicity of indoxyl sulfate and p-cresyl sulfate: a systematic review. Journal of the American Society of Nephrology: JASN. 2014;25(9):1897–907. 10.1681/ASN.2013101062 24812165 PMC4147984

[11] Sirich TL, Funk BA, Plummer NS, Hostetter TH, Meyer TW. Prominent accumulation in hemodialysis patients of solutes normally cleared by tubular secretion. Journal of the American Society of Nephrology: JASN. 2014;25(3):615–22. 10.1681/ASN.2013060597 24231664 PMC3935591

[12] Evans AM, DeHaven CD, Barrett T, Mitchell M, Milgram E. Integrated, nontargeted ultrahigh performance liquid chromatography/electrospray ionization tandem mass spectrometry platform for the identification and relative quantification of the small-molecule complement of biological systems. Anal Chem. 2009;81(16):6656–67. 10.1021/ac901536h 19624122

[13] Dehaven CD, Evans AM, Dai H, Lawton KA. Organization of GC/MS and LC/MS metabolomics data into chemical libraries. J Cheminform. 2010;2(1):9 10.1186/1758-2946-2-9 20955607 PMC2984397

[14] Caspi R, Altman T, Billington R, Dreher K, Foerster H, Fulcher CA, et al The MetaCyc database of metabolic pathways and enzymes and the BioCyc collection of Pathway/Genome Databases. Nucleic Acids Res. 2014;42(Database issue):D459–71. 10.1093/nar/gkt1103 24225315 PMC3964957

[15] Wishart DS, Jewison T, Guo AC, Wilson M, Knox C, Liu Y, et al HMDB 3.0—The Human Metabolome Database in 2013. Nucleic Acids Res. 2013;41(Database issue):D801–7. 10.1093/nar/gks1065 23161693 PMC3531200

[16] Consortium HM. A framework for human microbiome research. Nature. 2012;486(7402):215–21. 10.1038/nature11209 22699610 PMC3377744

[17] Vanholder R, De Smet R, Glorieux G, Argiles A, Baurmeister U, Brunet P, et al Review on uremic toxins: classification, concentration, and interindividual variability. Kidney Int. 2003;63(5):1934–43. doi: kid924 [pii] 10.1046/j.1523-1755.2003.00924.x 12675874

[18] Itoh Y, Ezawa A, Kikuchi K, Tsuruta Y, Niwa T. Protein-bound uremic toxins in hemodialysis patients measured by liquid chromatography/tandem mass spectrometry and their effects on endothelial ROS production. Anal Bioanal Chem. 2012;403(7):1841–50. 10.1007/s00216-012-5929-3 22447217

[19] Sirich TL, Aronov PA, Plummer NS, Hostetter TH, Meyer TW. Numerous protein-bound solutes are cleared by the kidney with high efficiency. Kidney Int. 2013;84(3):585–90. 10.1038/ki.2013.154 23636170 PMC3758437

[20] Niwa T, Yamamoto N, Maeda K, Yamada K, Ohki T, Mori M. Gas chromatographic—mass spectrometric analysis of polyols in urine and serum of uremic patients. Identification of new deoxyalditols and inositol isomers. J Chromatogr. 1983;277:25–39. 6643610

[21] Roboz J, Kappatos D, Holland JF. Polyol concentrations in serum during hemodialysis. Clin Chem. 1990;36(12):2082–6. 2253350

[22] Meyer TW, Hostetter TH. Uremic solutes from colon microbes. Kidney Int. 2012;81(10):949–54. 10.1038/ki.2011.504 22318422

[23] Mogk A, Bukau B. Cell biology. When the beginning marks the end. Science. 2010;327(5968):966–7. 10.1126/science.1187274 20167776

[24] Sass JO, Mohr V, Olbrich H, Engelke U, Horvath J, Fliegauf M, et al Mutations in ACY1, the gene encoding aminoacylase 1, cause a novel inborn error of metabolism. Am J Hum Genet. 2006;78(3):401–9. 10.1086/500563 16465618 PMC1380284

[25] Lindner HA, Tafler-Naumann M, Rohm KH. N-acetylamino acid utilization by kidney aminoacylase-1. Biochimie. 2008;90(5):773–80. 10.1016/j.biochi.2007.12.006 18222180

[26] Young VR, Alexis SD, Baliga BS, Munro HN, Muecke W. Metabolism of administered 3-methylhistidine. Lack of muscle transfer ribonucleic acid charging and quantitative excretion as 3-methylhistidine and its N-acetyl derivative. The Journal of biological chemistry. 1972;247(11):3592–600. 5030632

[27] Boldyrev AA, Aldini G, Derave W. Physiology and pathophysiology of carnosine. Physiol Rev. 2013;93(4):1803–45. 10.1152/physrev.00039.2012 24137022

[28] Sjolin J, Hjort G, Friman G, Hambraeus L. Urinary excretion of 1-methylhistidine: a qualitative indicator of exogenous 3-methylhistidine and intake of meats from various sources. Metabolism. 1987;36(12):1175–84. 3683186 10.1016/0026-0495(87)90245-9

[29] Raj DS, Ouwendyk M, Francoeur R, Pierratos A. Plasma amino acid profile on nocturnal hemodialysis. Blood Purif. 2000;18(2):97–102. doi: 14431. 10838467 10.1159/000014431

[30] Dou L, Sallee M, Cerini C, Poitevin S, Gondouin B, Jourde-Chiche N, et al The Cardiovascular Effect of the Uremic Solute Indole-3 Acetic Acid. Journal of the American Society of Nephrology: JASN. 2014 10.1681/ASN.2013121283 PMC4378098 25145928

[31] Biesaga M, Ochnik U, Pyrzynska K. Analysis of phenolic acids in fruits by HPLC with monolithic columns. J Sep Sci. 2007;30(17):2929–34. 10.1002/jssc.200700247 17960844

[32] Vine JH, Noiton D, Plummer JA, Baleriola-Lucas C, Mullins MG. Simultaneous quantitation of indole 3-acetic Acid and abscisic Acid in small samples of plant tissue by gas chromatography/mass spectrometry/selected ion monitoring. Plant Physiol. 1987;85(2):419–22. 16665713 10.1104/pp.85.2.419 PMC1054271

[33] Goldstein DS, Eisenhofer G, Kopin IJ. Sources and significance of plasma levels of catechols and their metabolites in humans. The Journal of pharmacology and experimental therapeutics. 2003;305(3):800–11. 10.1124/jpet.103.049270 12649306

[34] Uribarri J. The obsession with high dietary protein intake in ESRD patients on dialysis: is it justified? Nephron. 2000;86(2):105–8. doi: nef86105 [pii]. 11014977 10.1159/000045726

[35] Cathcart-Rake W, Porter R, Whittier F, Stein P, Carey M, Grantham J. Effect of diet on serum accumulation and renal excretion of aryl acids and secretory activity in normal and uremic man. Am J Clin Nutr. 1975;28(10):1110–5. 1180247 10.1093/ajcn/28.10.1110

[36] Crozier A, Jaganath IB, Clifford MN. Dietary phenolics: chemistry, bioavailability and effects on health. Nat Prod Rep. 2009;26(8):1001–43. 10.1039/b802662a 19636448

[37] Tsao R. Chemistry and biochemistry of dietary polyphenols. Nutrients. 2010;2(12):1231–46. 10.3390/nu2121231 22254006 PMC3257627

[38] Williamson G, Clifford MN. Colonic metabolites of berry polyphenols: the missing link to biological activity? The British journal of nutrition. 2010;104 Suppl 3:S48–66. 10.1017/S0007114510003946 20955650

[39] Cardona F, Andres-Lacueva C, Tulipani S, Tinahones FJ, Queipo-Ortuno MI. Benefits of polyphenols on gut microbiota and implications in human health. J Nutr Biochem. 2013;24(8):1415–22. 10.1016/j.jnutbio.2013.05.001 23849454

[40] Schepers E, Glorieux G, Vanholder R. The gut: the forgotten organ in uremia? Blood Purif. 2010;29(2):130–6. doi: 000245639 [pii] 10.1159/000245639 20093818

[41] Poesen R, Meijers B, Evenepoel P. The colon: an overlooked site for therapeutics in dialysis patients. Semin Dial. 2013;26(3):323–32. 10.1111/sdi.12082 23458264

[42] Wood A, Baxter G, Thies F, Kyle J, Duthie G. A systematic review of salicylates in foods: estimated daily intake of a Scottish population. Mol Nutr Food Res. 2011;55 Suppl 1:S7–S14. 10.1002/mnfr.201000408 21351247

[43] Clase CM, Ki V, Holden RM. Water-soluble vitamins in people with low glomerular filtration rate or on dialysis: a review. Semin Dial. 2013;26(5):546–67. 10.1111/sdi.12099 23859229 PMC4285924

[44] Tucker BM, Safadi S, Friedman AN. Is Routine Multivitamin Supplementation Necessary in US Chronic Adult Hemodialysis Patients? A Systematic Review. Journal of renal nutrition: the official journal of the Council on Renal Nutrition of the National Kidney Foundation. 2014 10.1053/j.jrn.2014.09.003 25446839

[45] Lindner A, Bankson DD, Stehman-Breen C, Mahuren JD, Coburn SP. Vitamin B6 metabolism and homocysteine in end-stage renal disease and chronic renal insufficiency. American journal of kidney diseases: the official journal of the National Kidney Foundation. 2002;39(1):134–45. 10.1053/ajkd.2002.29904 11774112

[46] Kalim S, Clish CB, Wenger J, Elmariah S, Yeh RW, Deferio JJ, et al A plasma long-chain acylcarnitine predicts cardiovascular mortality in incident dialysis patients. J Am Heart Assoc. 2013;2(6):e000542 10.1161/JAHA.113.000542 24308938 PMC3886735

[47] Fagugli RM, De Smet R, Buoncristiani U, Lameire N, Vanholder R. Behavior of non-protein-bound and protein-bound uremic solutes during daily hemodialysis. Am J Kidney Dis. 2002;40(2):339–47. 12148107 10.1053/ajkd.2002.34518

[48] Meyer TW, Leeper EC, Bartlett DW, Depner TA, Lit YZ, Robertson CR, et al Increasing dialysate flow and dialyzer mass transfer area coefficient to increase the clearance of protein-bound solutes. Journal of the American Society of Nephrology: JASN. 2004;15(7):1927–35. 15213283 10.1097/01.asn.0000131521.62256.f0

[49] Yeung CK, Shen DD, Thummel KE, Himmelfarb J. Effects of chronic kidney disease and uremia on hepatic drug metabolism and transport. Kidney Int. 2014;85(3):522–8. 10.1038/ki.2013.399 24132209 PMC4276411

[50] Psychogios N, Hau DD, Peng J, Guo AC, Mandal R, Bouatra S, et al The human serum metabolome. PLoS One. 2011;6(2):e16957 10.1371/journal.pone.0016957 21359215 PMC3040193

