# Supplementary material for: An Enlarged Profile of Uremic Solutes
Source: PLoS One. 2015 Aug 28;10(8):e0135657. doi: 10.1371/journal.pone.0135657 (PMC4552739; doi:10.1371/journal.pone.0135657)
Supplement: S1 Table — (DOC) [file pone.0135657.s001.doc]

**S1 Table. Characteristics of Hemodialysis Patients and Normal Subjects**

|  | Hemodialysis Patients (n=6) | Normal Subjects (n=6) |
| --- | --- | --- |
| Age (years) | 66 ± 14 | 43 ± 11 |
| Gender (f/m) | 0/6 | 2/4 |
| BMI (kg/m^2^) | 25 ± 4 | 27 ± 5 |
| Diabetes (yes/no) | 3/3 | 0/6 |
| Creatinine (mg/dl) | 9.2 ± 2.4 | 0.8 ± 0.2 |
| Dialysis Vintage (years) | 3.3 ± 2.3 | NA |
| Treatment Durations (hours) | 3.3 ± 0.5 | NA |
| Blood Flow Rate (ml/min) | 392 ± 20 | NA |
| Dialysate Flow Rate (ml/min) | 795 ± 8 | NA |
| Monthly Kt/V _urea_ | 1.68 ± 0.30 | NA |

Values shown are mean ± SD. NA, not applicable. Dialyzers used in hemodialysis patients were Revaclear (n=3) and Revaclear Max (n=3).
