# Supplementary material for: An Enlarged Profile of Uremic Solutes
Source: PLoS One. 2015 Aug 28;10(8):e0135657. doi: 10.1371/journal.pone.0135657 (PMC4552739; doi:10.1371/journal.pone.0135657)
Supplement: S2 Table — (DOC) [file pone.0135657.s002.doc]

**S2 Table. Uremic Solutes Found in the Present Study (n=120)**

| Solute | HMDB  Citation | Mass  Da | #HD/#Nl Total  Plasma | #HD/#Nl Plasma  UF | HD/Nl Total  Plasma | HD/Nl Plasma  UF | p-value  Total  Plasma | p-value  Plasma UF | q-value Total  Plasma | q-value  Plasma UF | Previously Identified as Uremic |
| --- | --- | --- | --- | --- | --- | --- | --- | --- | --- | --- | --- |
| Urea | HMDB00294 | 60 | 6/6 | 6/6 | 3 | 2 | 10^-2.7^ | 10^-2.3^ | 10^-2.2^ | 10^-2.1^ | x |
| Glycine | HMDB00123 | 75 | 6/6 | 6/6 | 3 | 2 | 10^-3.6^ | 10^-1.2^ | 10^-2.2^ | 10^-1.3^ | x |
| Beta-Alanine | HMDB00056 | 89 | 6/5 | 6/6 | 4 | 3 | 10^-1.8^ | 10^-1.3^ | 10^-1.8^ | 10^-1.4^ |  |
| 3-Aminoisobutanoic acid | HMDB03911 | 103 | 5/3 | 6/4 | 2 | 3 | 10^-1.0^ | 10^-1.7^ | 10^-1.2^ | 10^-1.8^ | x |
| Creatinine | HMDB00562 | 113 | 6/6 | 6/6 | 7 | 6 | 10^-6.7^ | 10^-6.4^ | 10^-2.2^ | 10^-2.2^ | x |
| Fumaric acid | HMDB00134 | 116 | 6/6 | 6/6 | 4 | 4 | 10^-6.9^ | 10^-4.2^ | 10^-2.2^ | 10^-2.2^ | x |
| D-Threitol | HMDB04136 | 122 | 6/6 | 6/6 | 18 | 5 | 10^-6.2^ | 10^-4.1^ | 10^-2.2^ | 10^-2.2^ | x |
| Erythritol | HMDB02994 | 122 | 6/6 | 6/6 | 4 | 3 | 10^-2.0^ | 10^-1.8^ | 10^-2.0^ | 10^-1.8^ | x |
| N-Acetyl-L-alanine | HMDB00766 | 131 | 6/6 | 6/6 | 6 | 4 | 10^-5.8^ | 10^-7.7^ | 10^-2.2^ | 10^-2.2^ | x |
| Erythronic acid | HMDB00613 | 136 | 6/6 | 6/6 | 21 | 18 | 10^-9.8^ | 10^-6.6^ | 10^-2.2^ | 10^-2.2^ | x |
| Threonic acid | HMDB00943 | 136 | 6/6 | 6/6 | 4 | 1 | 10^-2.0^ | 10^-0.4^ | 10^-1.9^ | 10^-0.7^ | x |
| Phenylacetic acid | HMDB00209 | 136 | 5/3 | 4/0 | 4 |  | 10^-2.4^ |  | 10^-2.1^ |  | x |
| 2-Aminobenzoic acid | HMDB01123 | 137 | 6/3 | 6/4 | 3 | 7 | 10^-1.3^ | 10^-2.5^ | 10^-1.4^ | 10^-2.2^ | x |
| Imidazolepropionic acid | HMDB02271 | 140 | 5/0 | 6/2 |  | 2 |  | 10^-0.4^ |  | 10^-0.7^ |  |
| Methylimidazoleacetic acid | HMDB02820 | 140 | 6/2 | 6/3 | 12 | 9 | 10^-1.6^ | 10^-4.4^ | 10^-1.6^ | 10^-2.2^ |  |
| Proline betaine | HMDB04827 | 143 | 6/6 | 6/6 | 3 | 3 | 10^-1.2^ | 10^-1.1^ | 10^-1.3^ | 10^-1.2^ | x |
| 4-Acetamidobutanoic acid | HMDB03681 | 145 | 6/6 | 6/6 | 19 | 26 | 10^-7.9^ | 10^-5.9^ | 10^-2.2^ | 10^-2.2^ | x |
| Isobutyrylglycine | HMDB00730 | 145 | 6/1 | 6/3 |  | 6 |  | 10^-2.2^ |  | 10^-2.1^ |  |
| 4-Guanidinobutanoic acid | HMDB03464 | 145 | 6/5 | 5/3 | 3 | 5 | 10^-2.4^ | 10^-1.7^ | 10^-2.1^ | 10^-1.8^ | x |
| N-Acetylserine | HMDB02931 | 147 | 6/6 | 6/6 | 14 | 2 | 10^-6.1^ | 10^-0.8^ | 10^-2.2^ | 10^-1.0^ |  |
| Tartaric acid | HMDB00956 | 150 | 6/1 | 6/3 |  | 38 |  | 10^-1.7^ |  | 10^-1.7^ |  |
| D-Xylose | HMDB00098 | 150 | 5/3 | 6/6 | 16 | 7 | 10^-1.8^ | 10^-2.1^ | 10^-1.8^ | 10^-2.0^ |  |
| L-Arabinose | HMDB00646 | 150 | 5/5 | 6/6 | 5 | 5 | 10^-3.0^ | 10^-3.8^ | 10^-2.2^ | 10^-2.2^ |  |
| p-Hydroxyphenylacetic acid | HMDB00020 | 152 | 6/3 | 6/6 | 66 | 70 | 10^-3.4^ | 10^-3.9^ | 10^-2.2^ | 10^-2.2^ | x |
| N1-Methyl-2-pyridone-5-carboxamide | HMDB04193 | 152 | 6/6 | 6/6 | 5 | 5 | 10^-4.2^ | 10^-3.9^ | 10^-2.2^ | 10^-2.2^ | x |
| Arabitol ^§^ | HMDB01851 | 152 | 6/6 | 6/6 | 10 | 9 | 10^-7.9^ | 10^-7.5^ | 10^-2.2^ | 10^-2.2^ | x |
| 2,5-Furandicarboxylic acid | HMDB04812 | 156 | 6/0 | 0/0 |  |  |  |  |  |  |  |
| Orotic acid | HMDB00226 | 156 | 5/3 | 6/3 | 5 | 8 | 10^-0.7^ | 10^-1.6^ | 10^-0.9^ | 10^-1.7^ | x |
| N-Acetylproline |  | 157 | 6/0 | 6/0 |  |  |  |  |  |  |  |
| Tiglylglycine | HMDB00959 | 157 | 6/0 | 6/1 |  |  |  |  |  |  | x |
| Allantoin | HMDB00462 | 158 | 6/6 | 6/6 | 15 | 12 | 10^-4.8^ | 10^-5.6^ | 10^-2.2^ | 10^-2.2^ | x |
| Isovalerylglycine | HMDB00678 | 159 | 6/0 | 6/5 |  | 12 |  | 10^-5.3^ |  | 10^-2.2^ | x |
| N-Acetylvaline | HMDB11757 | 159 | 6/3 | 6/6 | 3 | 2 | 10^-2.5^ | 10^-2.7^ | 10^-2.1^ | 10^-2.2^ |  |
| N-Acetylthreonine |  | 161 | 6/6 | 6/6 | 5 | 4 | 10^-7.5^ | 10^-6.0^ | 10^-2.2^ | 10^-2.2^ |  |
| Levoglucosan | HMDB00640 | 162 | 6/4 | 6/4 | 44 | 44 | 10^-2.8^ | 10^-3.1^ | 10^-2.2^ | 10^-2.2^ |  |
| L-Fucose | HMDB00174 | 164 | 5/5 | 5/6 | 3 | 5 | 10^-2.1^ | 10^-2.9^ | 10^-2.0^ | 10^-2.2^ |  |
| Arabinonic acid | HMDB00539 | 166 | 6/6 | 6/6 | 16 | 6 | 10^-7.6^ | 10^-5.2^ | 10^-2.2^ | 10^-2.2^ | x |
| L-Xylonate | HMDB60256 | 166 | 6/6 | 6/6 | 12 | 26 | 10^-4.3^ | 10^-4.7^ | 10^-2.2^ | 10^-2.2^ |  |
| Quinolinic acid | HMDB00232 | 167 | 6/4 | 6/1 | 10 |  | 10^-3.8^ |  | 10^-2.2^ |  | x |
| Vanillic acid | HMDB00484 | 168 | 6/3 | 6/1 | 6 |  | 10^-3.6^ |  | 10^-2.2^ |  | x |
| 2-Furoylglycine | HMDB00439 | 169 | 6/1 | 6/1 |  |  |  |  |  |  | x |
| 1-Methylhistidine | HMDB00001 | 169 | 6/2 | 0/0 | 7 |  | 10^-2.4^ |  | 10^-2.1^ |  | x |
| 3-Methylhistidine | HMDB00479 | 169 | 6/5 | 6/6 | 3 | 3 | 10^-0.8^ | 10^-1.3^ | 10^-1.1^ | 10^-1.5^ | x |
| Phenol sulphate | HMDB60015 | 174 | 6/6 | 6/6 | 8 | 13 | 10^-3.0^ | 10^-3.6^ | 10^-2.2^ | 10^-2.2^ | x |
| Indoleacetic acid | HMDB00197 | 175 | 6/5 | 6/2 | 4 | 12 | 10^-1.9^ | 10^-2.4^ | 10^-1.9^ | 10^-2.1^ | x |
| Citrulline | HMDB00904 | 175 | 6/6 | 6/6 | 3 | 2 | 10^-2.8^ | 10^-2.8^ | 10^-2.2^ | 10^-2.2^ | x |
| N-Formyl-L-methionine | HMDB01015 | 177 | 6/6 | 6/6 | 3 | 3 | 10^-5.1^ | 10^-5.2^ | 10^-2.2^ | 10^-2.2^ |  |
| L-Gulonolactone | HMDB03466 | 178 | 6/6 | 6/6 | 22 | 25 | 10^-7.0^ | 10^-4.8^ | 10^-2.2^ | 10^-2.2^ |  |
| Hippuric acid | HMDB00714 | 179 | 6/6 | 6/6 | 21 | 32 | 10^-4.2^ | 10^-4.9^ | 10^-2.2^ | 10^-2.2^ | x |
| Levoinositol | HMDB34220 | 180 | 6/3 | 6/3 | 16 | 24 | 10^-2.0^ | 10^-3.0^ | 10^-2.0^ | 10^-2.2^ | x |
| Myoinositol | HMDB00211 | 180 | 6/6 | 6/6 | 11 | 6 | 10^-9.5^ | 10^-6.2^ | 10^-2.2^ | 10^-2.2^ | x |
| Scyllitol | HMDB06088 | 180 | 6/6 | 6/6 | 5 | 3 | 10^-3.1^ | 10^-2.3^ | 10^-2.2^ | 10^-2.1^ | x |
| 7-Methyluric acid | HMDB11107 | 182 | 5/2 | 5/3 | 40 | 49 | 10^-1.7^ | 10^-3.2^ | 10^-1.8^ | 10^-2.2^ |  |
| 1-Methyluric acid | HMDB03099 | 182 | 6/6 | 6/4 | 12 | 11 | 10^-3.6^ | 10^-2.8^ | 10^-2.2^ | 10^-2.2^ | x |
| Galactitol | HMDB00107 | 182 | 6/0 | 6/1 |  |  |  |  |  |  |  |
| Mannitol | HMDB00765 | 182 | 6/6 | 6/6 | 49 | 36 | 10^-3.5^ | 10^-3.4^ | 10^-2.2^ | 10^-2.2^ | x |
| Saccharin | HMDB29723 | 183 | 6/2 | 6/1 | 22 |  | 10^-0.6^ |  | 10^-0.9^ |  |  |
| 4-Pyridoxic acid | HMDB00017 | 183 | 6/6 | 6/3 | 74 | 93 | 10^-3.4^ | 10^-3.1^ | 10^-2.2^ | 10^-2.2^ | x |
| Acisoga |  | 184 | 6/6 | 6/6 | 3 | 3 | 10^-3.6^ | 10^-3.6^ | 10^-2.2^ | 10^-2.2^ |  |
| p-Cresol sulfate | HMDB11635 | 188 | 6/6 | 6/6 | 13 | 21 | 10^-3.5^ | 10^-4.0^ | 10^-2.2^ | 10^-2.2^ | x |
| 2-Aminophenol sulphate | HMDB61116 | 189 | 6/6 | 6/6 | 8 | 10 | 10^-3.4^ | 10^-3.9^ | 10^-2.2^ | 10^-2.2^ |  |
| Kynurenic acid | HMDB00715 | 189 | 6/6 | 6/0 | 7 |  | 10^-4.5^ |  | 10^-2.2^ |  | x |
| Indole-3-methyl acetate | HMDB29738 | 189 | 6/0 | 6/0 |  |  |  |  |  |  |  |
| Homocitrulline | HMDB00679 | 189 | 6/5 | 6/6 | 5 | 7 | 10^-4.2^ | 10^-3.7^ | 10^-2.2^ | 10^-2.2^ | x |
| Pyrocatechol sulfate | HMDB59724 | 190 | 6/6 | 6/6 | 3 | 3 | 10^-2.4^ | 10^-2.5^ | 10^-2.1^ | 10^-2.2^ | x |
| 2-Oxindole-3-acetate |  | 191 | 5/0 | 6/0 |  |  |  |  |  |  |  |
| N-Acetyl-L-methionine | HMDB11745 | 191 | 6/6 | 6/6 | 8 | 6 | 10^-6.1^ | 10^-5.6^ | 10^-2.2^ | 10^-2.2^ |  |
| Phenylacetylglycine | HMDB00821 | 193 | 6/0 | 6/1 |  |  |  |  |  |  | x |
| 4-Hydroxyhippuric acid | HMDB13678 | 195 | 6/6 | 6/4 | 66 | 99 | 10^-6.7^ | 10^-6.5^ | 10^-2.2^ | 10^-2.2^ | x |
| Salicyluric acid | HMDB00840 | 195 | 6/4 | 6/1 | 49 |  | 10^-1.4^ |  | 10^-1.5^ |  | x |
| 3-Hydroxyhippuric acid | HMDB06116 | 195 | 6/6 | 6/6 | 7 | 9 | 10^-3.1^ | 10^-3.2^ | 10^-2.2^ | 10^-2.2^ | x |
| Gluconic acid | HMDB00625 | 196 | 6/6 | 6/6 | 21 | 18 | 10^-6.6^ | 10^-5.2^ | 10^-2.2^ | 10^-2.2^ | x |
| 1,7-Dimethyluric acid | HMDB11103 | 196 | 6/4 | 6/4 | 4 | 5 | 10^-1.3^ | 10^-1.6^ | 10^-1.4^ | 10^-1.7^ | x |
| N-Acetylhistidine | HMDB32055 | 197 | 6/2 | 6/5 | 3 | 5 | 10^-2.2^ | 10^-4.3^ | 10^-2.0^ | 10^-2.2^ |  |
| Vanillylmandelic acid | HMDB00291 | 198 | 6/5 | 6/5 | 15 | 24 | 10^-6.3^ | 10^-4.6^ | 10^-2.2^ | 10^-2.2^ | x |
| 4-Vinylphenol sulfate |  | 200 | 6/6 | 6/0 | 11 |  | 10^-4.5^ |  | 10^-2.2^ |  |  |
| 2-Methoxyphenol sulfate |  | 204 | 6/6 | 6/6 | 25 | 41 | 10^-4.1^ | 10^-3.2^ | 10^-2.2^ | 10^-2.2^ |  |
| 3-Methylcatechol sulfate ^†^ |  | 204 | 6/5 | 5/0 | 5 |  | 10^-2.2^ |  | 10^-2.1^ |  |  |
| 4-Methylcatechol sulfate |  | 204 | 6/6 | 6/5 | 3 | 8 | 10^-1.2^ | 10^-2.4^ | 10^-1.3^ | 10^-2.1^ | x |
| Cinnamoylglycine | HMDB11621 | 205 | 6/4 | 6/0 | 12 |  | 10^-2.0^ |  | 10^-1.9^ |  | x |
| Indolelactic acid | HMDB00671 | 205 | 6/6 | 6/1 | 3 |  | 10^-1.3^ |  | 10^-1.4^ |  | x |
| N-Acetyl-L-phenylalanine | HMDB00512 | 207 | 6/3 | 6/6 | 3 | 5 | 10^-2.2^ | 10^-4.3^ | 10^-2.1^ | 10^-2.2^ |  |
| 1,3,7-Trimethyluric acid | HMDB02123 | 210 | 6/5 | 6/3 | 4 | 3 | 10^-1.9^ | 10^-1.6^ | 10^-1.9^ | 10^-1.7^ | x |
| N-acetyl-3-methylhistidine* |  | 211 | 6/4 | 6/3 | 10 | 14 | 10^-3.0^ | 10^-3.3^ | 10^-2.2^ | 10^-2.2^ |  |
| N-acetyl-1-methylhistidine* |  | 211 | 6/4 | 6/4 | 7 | 4 | 10^-3.5^ | 10^-1.8^ | 10^-2.2^ | 10^-1.8^ |  |
| Indoxyl sulfate | HMDB00682 | 213 | 6/6 | 6/6 | 7 | 20 | 10^-5.3^ | 10^-5.7^ | 10^-2.2^ | 10^-2.2^ | x |
| N2,N5-diacetylornithine |  | 216 | 5/3 | 6/6 | 4 | 4 | 10^-2.3^ | 10^-3.3^ | 10^-2.1^ | 10^-2.2^ |  |
| N-Acetyl alliin* |  | 219 | 4/5 | 5/5 |  | 8 | 10^-2.3^ | 10^-1.4^ | 10^-2.1^ | 10^-1.5^ |  |
| Pantothenic acid | HMDB00210 | 219 | 6/6 | 6/6 | 8 | 7 | 10^-2.3^ | 10^-2.4^ | 10^-2.1^ | 10^-2.1^ | x |
| Pyroglutamylvaline |  | 228 | 6/0 | 6/0 |  |  |  |  |  |  |  |
| Prolylhydroxyproline | HMDB06695 | 228 | 6/6 | 6/6 | 9 | 10 | 10^-5.1^ | 10^-5.5^ | 10^-2.2^ | 10^-2.2^ | x |
| Isobutyryl-L-carnitine | HMDB00736 | 231 | 6/6 | 6/6 | 5 | 4 | 10^-1.6^ | 10^-1.2^ | 10^-1.7^ | 10^-1.3^ |  |
| Phenylcarnitine* |  | 237 | 6/1 | 6/0 |  |  |  |  |  |  |  |
| Cytidine | HMDB00089 | 243 | 6/4 | 6/5 | 4 | 3 | 10^-6.4^ | 10^-4.6^ | 10^-2.2^ | 10^-2.2^ | x |
| Pseudouridine | HMDB00767 | 244 | 6/6 | 6/6 | 7 | 5 | 10^-6.4^ | 10^-5.7^ | 10^-2.2^ | 10^-2.2^ | x |
| 3-[3-(sulfooxy)phenyl]propanoic acid | | 246 | 6/2 | 6/2 | 13 | 44 | 10^-2.3^ | 10^-3.8^ | 10^-2.1^ | 10^-2.2^ |  |
| N-Acetyltryptophan | HMDB13713 | 246 | 6/1 | 6/0 |  |  |  |  |  |  | x |
| 2-Hydroxyacetaminophen sulfate* |  | 247 | 5/1 | 4/1 |  |  |  |  |  |  |  |
| Gamma-CEHC | HMDB01931 | 248 | 6/6 | 2/0 | 4 |  | 10^-2.3^ |  | 10^-2.1^ |  | x |
| L-gamma-glutamyl-L-isoleucine | HMDB11170 | 260 | 6/6 | 6/6 | 3 | 4 | 10^-3.4^ | 10^-4.8^ | 10^-2.2^ | 10^-2.2^ |  |
| O-sulfo-L-tyrosine |  | 261 | 6/5 | 6/6 | 11 | 11 | 10^-6.2^ | 10^-4.3^ | 10^-2.2^ | 10^-2.2^ |  |
| Homovanillic acid sulfate | HMDB11719 | 262 | 6/0 | 6/1 |  |  |  |  |  |  |  |
| Alpha-N-Phenylacetyl-L-glutamine | HMDB06344 | 264 | 6/6 | 6/6 | 44 | 32 | 10^-6.3^ | 10^-6.1^ | 10^-2.2^ | 10^-2.2^ | x |
| Acetylcarnosine | HMDB12881 | 268 | 6/6 | 6/6 | 6 | 5 | 10^-4.4^ | 10^-4.4^ | 10^-2.2^ | 10^-2.2^ |  |
| Glutarylcarnitine | HMDB13130 | 275 | 6/5 | 0/0 | 5 |  | 10^-5.6^ |  | 10^-2.2^ |  | x |
| 3-Methylglutarylcarnitine | HMDB00552 | 289 | 6/5 | 6/6 | 37 | 31 | 10^-7.2^ | 10^-8.2^ | 10^-2.2^ | 10^-2.2^ | x |
| 5'-Methylthioadenosine | HMDB01173 | 297 | 6/3 | 6/6 | 3 | 4 | 10^-1.0^ | 10^-2.9^ | 10^-1.2^ | 10^-2.2^ |  |
| Indoleacetyl glutamine | HMDB13240 | 303 | 6/0 | 6/2 |  | 67 |  | 10^-4.4^ |  | 10^-2.2^ |  |
| N-Acetylneuraminic acid | HMDB00230 | 309 | 5/4 | 6/6 | 8 | 7 | 10^-2.0^ | 10^-2.6^ | 10^-1.9^ | 10^-2.2^ | x |
| N2,N2-Dimethylguanosine | HMDB04824 | 311 | 6/6 | 6/6 | 12 | 11 | 10^-5.2^ | 10^-6.5^ | 10^-2.2^ | 10^-2.2^ | x |
| Sucrose | HMDB00258 | 342 | 6/1 | 6/5 |  | 35 |  | 10^-4.1^ |  | 10^-2.2^ | x |
| C-mannosyltryptophan |  | 366 | 6/6 | 6/6 | 16 | 13 | 10^-6.3^ | 10^-6.9^ | 10^-2.2^ | 10^-2.2^ | x |
| Androsterone sulfate | HMDB02759 | 370 | 6/6 | 4/1 | 3 |  | 10^-1.8^ |  | 10^-1.8^ |  | x |
| Salicyluric glucuronide* |  | 371 | 6/3 | 6/0 | 315 |  | 10^-2.4^ |  | 10^-2.1^ |  | x |
| Riboflavin | HMDB00244 | 376 | 5/1 | 5/2 |  | 12 |  | 10^-1.8^ |  | 10^-1.8^ | x |
| S-Adenosylhomocysteine | HMDB00939 | 384 | 6/0 | 6/1 |  |  |  |  |  |  | x |
| N6-Carbamoyl-L-threonyladenosine | HMDB41623 | 412 | 6/6 | 6/6 | 24 | 20 | 10^-6.3^ | 10^-6.4^ | 10^-2.2^ | 10^-2.2^ | x |
| Gamma-CEHC glucuronide* |  | 424 | 6/1 | 6/1 |  |  |  |  |  |  |  |
| Alpha-CEHC glucuronide* |  | 454 | 6/0 | 6/0 |  |  |  |  |  |  |  |
| 6-Sialyl-N-acetyllactosamine | HMDB06584 | 674 | 6/1 | 6/2 |  | 30 |  | 10^-6.2^ |  | 10^-2.2^ |  |

Total 120 uremic solutes are listed in order of increasing molecular mass. #HD / #Nl represents the numbers that we detected the solute in plasma or plasma ultrafiltrate of HD patients or normal subjects. HD / Nl represents concentration ratios predialysis compared to normal in plasma or plasma ultrafiltrate, as estimated from mass spectrometric peak areas. " Previously Identified as Uremic " indicates solutes that had previously been reported to accumulate in renal failure. * indicates a solute for which a reagent standard was not run but for which identity was considered well established by MS/MS. ^§^ indicates that the analysis does not distinguish between the D- and L- forms of arabitol. ^†^ indicates that the analytic method did not distinguish which OH group on 3-Methylcatehol had been sulfated.
