# Supplementary material for: An Enlarged Profile of Uremic Solutes
Source: PLoS One. 2015 Aug 28;10(8):e0135657. doi: 10.1371/journal.pone.0135657 (PMC4552739; doi:10.1371/journal.pone.0135657)
Supplement: S3 Table — (DOC) [file pone.0135657.s003.doc]

**S3 Table. Solutes Classified as Uremic on Original Analysis Which Were Not**

**Detected When Samples from Dialysis Patients With and Without Colons Were Compared**

| Solute | HMDB Citation | Mass Da |
| --- | --- | --- |
| 2-Aminobenzoic acid | HMDB01123 | 137 |
| D-Xylose | HMDB00098 | 150 |
| 2,5-Furandicarboxylic acid | HMDB04812 | 156 |
| N-Acetylproline |  | 157 |
|  |  |  |
| L-Gulonolactone | HMDB03466 | 178 |
| 7-Methyluric acid | HMDB11107 | 182 |
| Galactitol | HMDB00107 | 182 |
| Saccharin | HMDB29723 | 183 |
| N2,N5-diacetylornithine |  | 216 |
| N-Acetyl alliin* |  | 219 |
| 2-hydroxyacetaminophen sulfate |  | 247 |
| Homovanillic acid sulfate | HMDB11719 | 262 |

Of the 120 solutes categorized as uremic in our initial analysis were not detected in the samples from dialysis patients with and without colons analyzed using the newer Metabolon platform, and the question whether these solutes were colon-derived was thus not addressed. * indicates a solute for which a reagent standard was not run but for which identity was considered well established by MS/MS
