# Supplementary material for: An Enlarged Profile of Uremic Solutes
Source: PLoS One. 2015 Aug 28;10(8):e0135657. doi: 10.1371/journal.pone.0135657 (PMC4552739; doi:10.1371/journal.pone.0135657)
Supplement: S4 Table — (DOC) [file pone.0135657.s004.doc]

**S4 Table. Uremic Solutes Found in the Literature and Uremic Solutes Found in the Present Study (n=278)**

| Exact Mass | Solute | HMDB Citation | Present Study | References |
| --- | --- | --- | --- | --- |
| 31.0422 | Methylamine | HMDB00164 |  |  |
| 45.0578 | Dimethylamine | HMDB00087 |  |  |
| 45.0578 | Ethylamine | HMDB13231 |  |  |
| 56.0262 | Acrolein | HMDB41822 |  |  |
| 57.9751 | Thiocyanate | HMDB01453 |  |  |
| 58.0055 | Glyoxal |  |  |  |
| 59.0483 | Guanidine | HMDB01842 |  |  |
| 59.0735 | Trimethylamine | HMDB00906 |  |  |
| 60.0324 | Urea | HMDB00294 | Yes |  |
| 72.0211 | Malondialdehyde | HMDB06112 |  |  |
| 72.0211 | Pyruvaldehyde | HMDB01167 |  |  |
| 73.0640 | Methylguanidine | HMDB01522 |  |  |
| 75.0320 | Glycine | HMDB00123 | Yes |  |
| 75.0684 | Trimethylamine N-oxide | HMDB00925 |  |  |
| 88.1000 | Putrescine | HMDB01414 |  |  |
| 89.0113 | Oxamate |  |  |  |
| 89.0477 | Beta-Alanine | HMDB00056 | Yes | novel |
| 89.9953 | Oxalic acid | HMDB02329 |  |  |
| 94.0419 | Phenol | HMDB00228 |  |  |
| 98.0732 | 2-Hexenal | HMDB31496 |  |  |
| 100.0888 | Hexanal | HMDB05994 |  |  |
| 103.0633 | Dimethylglycine | HMDB00092 |  |  |
| 103.0633 | 3-Aminoisobutanoic acid | HMDB03911 | Yes |  |
| 103.0633 | 2-Aminoisobutyric acid | HMDB01906 |  |  |
| 104.0110 | Malonic acid | HMDB00691 |  |  |
| 104.1075 | Choline | HMDB00097 |  |  |
| 104.0473 | Alpha-Hydroxyisobutyric acid | HMDB00729 |  |  |
| 108.0575 | Benzyl alcohol | HMDB03119 |  |  |
| 108.0575 | p-Cresol | HMDB01858 |  |  |
| 109.0197 | Hypotaurine | HMDB00965 |  |  |
| 110.0368 | Hydroquinone | HMDB02434 |  |  |
| 110.0368 | Pyrocatechol | HMDB00957 |  |  |
| 111.0433 | Cytosine | HMDB00630 |  |  |
| 112.0273 | Uracil | HMDB00300 |  |  |
| 112.0888 | 2-Heptenal | HMDB33827 |  |  |
| 113.0589 | Creatinine | HMDB00562 | Yes |  |
| 114.0681 | 4-HO-hexenal |  |  |  |
| 114.1045 | Heptanal | HMDB31475 |  |  |
| 116.0110 | Fumaric acid | HMDB00134 | Yes |  |
| 116.0110 | Maleic acid | HMDB00176 |  |  |
| 116.0473 | Levulinic acid | HMDB00720 |  |  |
| 117.0538 | Guanidoacetic acid | HMDB00128 |  |  |
| 118.0266 | Methylmalonic acid | HMDB00202 |  |  |
| 118.0266 | Succinic acid | HMDB00254 |  |  |
| 118.0630 | 2-Ethylhydracrylic acid | HMDB00396 |  |  |
| 121.0197 | D-Cysteine | HMDB03417 |  |  |
| 122.0480 | Niacinamide | HMDB01406 |  |  |
| 122.0579 | D-Threitol | HMDB04136 | Yes |  |
| 122.0579 | Erythritol | HMDB02994 | Yes |  |
| 125.9987 | 2-Hydroxyethanesulfonate | HMDB03903 |  |  |
| 126.0429 | Thymine | HMDB00262 |  |  |
| 126.1045 | 2-Octenal | HMDB30961 |  |  |
| 128.1292 | Pyroglutamine |  |  |  |
| 129.0426 | Pyroglutamic acid | HMDB00267 |  |  |
| 130.0266 | Citraconic acid | HMDB00634 |  |  |
| 131.0582 | Hydroxyproline | HMDB00725 |  |  |
| 131.0582 | N-Acetyl-L-alanine | HMDB00766 | Yes |  |
| 131.0695 | Beta-Guanidinopropionic acid | HMDB13222 |  |  |
| 131.0695 | Creatine | HMDB00064 |  |  |
| 132.0423 | Glutaric acid | HMDB00661 |  |  |
| 132.0423 | Methylsuccinic acid | HMDB01844 |  |  |
| 132.0535 | Ureidopropionic acid | HMDB00026 |  |  |
| 133.0528 | 5-Hydroxyindole | HMDB59805 |  |  |
| 135.0354 | Homocysteine | HMDB00742 |  |  |
| 135.0545 | Adenine | HMDB00034 |  |  |
| 136.0372 | Erythronic acid | HMDB00613 | Yes |  |
| 136.0372 | Threonic acid | HMDB00943 | Yes |  |
| 136.0385 | Hypoxanthine | HMDB00157 |  |  |
| 136.0524 | Phenylacetic acid | HMDB00209 | Yes |  |
| 137.0477 | 2-Aminobenzoic acid | HMDB01123 | Yes |  |
| 138.0317 | Salicylic acid | HMDB01895 |  |  |
| 138.0317 | 4-Hydroxybenzoic acid | HMDB00500 |  |  |
| 138.0317 | 3-Hydroxybenzoic acid | HMDB02466 |  |  |
| 140.0473 | 2-Methoxyresorcinol |  |  |  |
| 140.0586 | Imidazolepropionic acid | HMDB02271 | Yes | novel |
| 140.0586 | Methylimidazoleacetic acid | HMDB02820 | Yes | novel |
| 140.1201 | 2-Nonenal | HMDB31269 |  |  |
| 142.0990 | 4-HO-octenal |  |  |  |
| 142.1358 | Nonanal | HMDB59835 |  |  |
| 143.0946 | Proline betaine | HMDB04827 | Yes |  |
| 145.0739 | 4-Acetamidobutanoic acid | HMDB03681 | Yes |  |
| 145.0739 | Isobutyrylglycine | HMDB00730 | Yes | novel |
| 145.0851 | 4-Guanidinobutanoic acid | HMDB03464 | Yes |  |
| 145.1579 | Spermidine | HMDB01257 |  |  |
| 146.0579 | Adipic acid | HMDB00448 |  |  |
| 146.1181 | 4-Trimethylammoniobutanoic acid | HMDB01161 |  |  |
| 147.0532 | N-Acetylserine | HMDB02931 | Yes | novel |
| 148.0372 | Citramalic acid | HMDB00426 |  |  |
| 150.0164 | Tartaric acid | HMDB00956 | Yes | novel |
| 150.0528 | D-Xylose | HMDB00098 | Yes | novel |
| 150.0528 | L-Arabinose | HMDB00646 | Yes | novel |
| 152.0334 | Xanthine | HMDB00292 |  |  |
| 152.0473 | p-Hydroxyphenylacetic acid | HMDB00020 | Yes |  |
| 152.0473 | 3-Hydroxyphenylacetic acid | HMDB00440 |  |  |
| 152.0473 | Ortho-Hydroxyphenylacetic acid | HMDB00669 |  |  |
| 152.0586 | N1-Methyl-2-pyridone-5-carboxamide | HMDB04193 | Yes |  |
| 152.0586 | N1-Methyl-4-pyridone-3-carboxamide | HMDB04194 |  |  |
| 152.0685 | D-Arabitol/L-Arabitol^§^ | HMDB01851 | Yes |  |
| 154.1358 | 4-Decenal | HMDB41014 |  |  |
| 156.0059 | 2,5-Furandicarboxylic acid | HMDB04812 | Yes | novel |
| 156.0171 | Orotic acid | HMDB00226 | Yes |  |
| 156.1150 | 4-Hydroxynonenal | HMDB04362 |  |  |
| 156.1514 | Decanal | HMDB11623 |  |  |
| 157.0739 | Tiglylglycine | HMDB00959 | Yes |  |
| 157.0739 | N-acetylproline |  | Yes | novel |
| 158.0440 | Allantoin | HMDB00462 | Yes |  |
| 159.0895 | N-Acetylvaline | HMDB11757 | Yes | novel |
| 159.0895 | Isovalerylglycine | HMDB00678 | Yes |  |
| 160.0736 | Pimelic acid | HMDB00857 |  |  |
| 161.0688 | N-Acetylthreonine |  | Yes | novel |
| 162.0528 | Levoglucosan | HMDB00640 | Yes | novel |
| 162.0528 | 3-Deoxyglucosone |  |  |  |
| 164.0685 | L-Fucose | HMDB00174 | Yes | novel |
| 165.0460 | Methionine sulfoxide | HMDB02005 |  |  |
| 165.0651 | 7-Methylguanine | HMDB00897 |  |  |
| 166.0266 | Phthalic acid | HMDB02107 |  |  |
| 166.0477 | Arabinonic acid | HMDB00539 | Yes |  |
| 166.0477 | L-Xylonate | HMDB60256 | Yes | novel |
| 166.0491 | 3-Methylxanthine | HMDB01886 |  |  |
| 167.0219 | Quinolinic acid | HMDB00232 | Yes |  |
| 167.0365 | Taurocyamine | HMDB03584 |  |  |
| 168.0283 | Uric acid | HMDB00289 |  |  |
| 168.0423 | Homogentisic acid | HMDB00130 |  |  |
| 168.0423 | Vanillic acid | HMDB00484 | Yes |  |
| 169.0375 | 2-Furoylglycine | HMDB00439 | Yes |  |
| 169.0739 | Norepinephrine | HMDB00216 |  |  |
| 169.0851 | 1-Methylhistidine | HMDB00001 | Yes |  |
| 169.0851 | 3-Methylhistidine | HMDB00479 | Yes |  |
| 170.1307 | 4-HO-decenal |  |  |  |
| 173.0800 | 2-Oxoarginine | HMDB04225 |  |  |
| 173.9119 | Pyrophosphate | HMDB00250 |  |  |
| 173.9987 | Phenol sulphate | HMDB60015 | Yes |  |
| 174.0164 | cis-Aconitic acid | HMDB00072 |  |  |
| 174.0164 | trans-Aconitic acid | HMDB00958 |  |  |
| 174.0892 | Suberic acid | HMDB00893 |  |  |
| 174.1004 | N-Acetylornithine | HMDB03357 |  |  |
| 175.0481 | N-Acetyl-L-aspartic acid | HMDB00812 |  |  |
| 175.0593 | Guanidinosuccinic acid | HMDB03157 |  |  |
| 175.0633 | Indoleacetic acid | HMDB00197 | Yes |  |
| 175.0957 | Argininic acid | HMDB03148 |  |  |
| 175.0957 | Citrulline | HMDB00904 | Yes |  |
| 176.0685 | 2-Isopropylmalic acid | HMDB00402 |  |  |
| 177.0460 | N-Formyl-L-methionine | HMDB01015 | Yes | novel |
| 177.0790 | 5-Hydroxytryptophol | HMDB01855 |  |  |
| 178.0477 | L-Gulonolactone | HMDB03466 | Yes | novel |
| 179.0582 | Hippuric acid | HMDB00714 | Yes |  |
| 180.0634 | Myoinositol | HMDB00211 | Yes |  |
| 180.0634 | Levoinositol | HMDB34220 | Yes |  |
| 180.0634 | Scyllitol | HMDB06088 | Yes |  |
| 182.0440 | 1-Methyluric acid | HMDB03099 | Yes |  |
| 182.0440 | 7-Methyluric acid | HMDB11107 | Yes | novel |
| 182.0579 | Hydroxyphenyllactic acid | HMDB00755 |  |  |
| 182.0579 | Homovanillic acid | HMDB00118 |  |  |
| 182.0790 | Mannitol | HMDB00765 | Yes |  |
| 182.0790 | Sorbitol | HMDB00247 |  |  |
| 182.0790 | Galactitol | HMDB00107 | Yes | novel |
| 182.9990 | Saccharin | HMDB29723 | Yes | novel |
| 183.0532 | 4-Pyridoxic acid | HMDB00017 | Yes |  |
| 184.1212 | Acisoga |  | Yes | novel |
| 188.0143 | p-Cresol sulfate | HMDB11635 | Yes |  |
| 188.1049 | Azelaic acid | HMDB00784 |  |  |
| 188.1161 | N6-Acetyl-L-lysine | HMDB00206 |  |  |
| 188.1525 | N6,N6,N6-Trimethyl-L-lysine | HMDB01325 |  |  |
| 189.0096 | 2-Aminophenol sulphate | HMDB61116 | Yes | novel |
| 189.0426 | Kynurenic acid | HMDB00715 | Yes |  |
| 189.0637 | N-Acetylglutamic acid | HMDB01138 |  |  |
| 189.0790 | Indole-3-methyl acetate | HMDB29738 | Yes | novel |
| 189.1113 | Homocitrulline | HMDB00679 | Yes |  |
| 189.9936 | Hydroquinone sulfate |  |  |  |
| 189.9936 | Pyrocatechol sulfate | HMDB59724 | Yes |  |
| 190.0504 | 2-Oxindole-3-acetate |  | Yes | novel |
| 191.0582 | 5-Hydroxyindoleacetic acid | HMDB00763 |  |  |
| 191.0616 | N-Acetyl-L-methionine | HMDB11745 | Yes | novel |
| 192.0270 | Isocitric acid | HMDB00193 |  |  |
| 192.0270 | Citric acid | HMDB00094 |  |  |
| 192.0634 | Quinic acid | HMDB03072 |  |  |
| 193.0739 | Phenylacetylglycine | HMDB00821 | Yes |  |
| 195.0532 | 4-Hydroxyhippuric acid | HMDB13678 | Yes |  |
| 195.0532 | Salicyluric acid | HMDB00840 | Yes |  |
| 195.0532 | 3-Hydroxyhippuric acid | HMDB06116 | Yes |  |
| 196.0583 | Gluconic acid | HMDB00625 | Yes |  |
| 196.0596 | 1,3-Dimethyluric acid | HMDB01857 |  |  |
| 196.0596 | 1,7-Dimethyluric acid | HMDB11103 | Yes |  |
| 197.0688 | L-Dopa | HMDB00181 |  |  |
| 197.0800 | N-Acetylhistidine | HMDB32055 | Yes | novel |
| 198.0528 | Vanillylmandelic acid | HMDB00291 | Yes |  |
| 198.0753 | 5-Acetylamino-6-amino-3-methyluracil | HMDB04400 |  |  |
| 200.0143 | 4-Vinylphenol sulfate |  | Yes | novel |
| 200.9766 | Cysteine-S-sulfate | HMDB00731 |  |  |
| 202.0300 | 4-Ethylphenyl sulfate |  |  |  |
| 202.1205 | Sebacic acid | HMDB00792 |  |  |
| 202.1430 | Asymmetric dimethylarginine | HMDB01539 |  |  |
| 202.1430 | Symmetric dimethylarginine | HMDB03334 |  |  |
| 202.2157 | Spermine | HMDB01256 |  |  |
| 204.0092 | 3-Methylcatechol sulfate ^†^ |  | Yes | novel |
| 204.0092 | 2-Methoxyphenol sulfate |  | Yes | novel |
| 204.0092 | 4-Methylcatechol sulfate |  | Yes |  |
| 204.1110 | Nξ-(carboxymethyl)lysine |  |  |  |
| 205.0375 | Xanthurenic acid | HMDB00881 |  |  |
| 205.0739 | Cinnamoylglycine | HMDB11621 | Yes |  |
| 205.0739 | Indolelactic acid | HMDB00671 | Yes |  |
| 207.0895 | N-Acetyl-L-phenylalanine | HMDB00512 | Yes | novel |
| 208.0848 | L-Kynurenine | HMDB00684 |  |  |
| 210.0753 | 1,3,7-Trimethyluric acid | HMDB02123 | Yes |  |
| 211.0957 | N-Acetyl-1-methylhistidine* |  | Yes | novel |
| 211.0957 | N-Acetyl-3-methylhistidine* |  | Yes | novel |
| 213.0096 | Indoxyl sulfate | HMDB00682 | Yes |  |
| 216.1110 | N2,N5-diacetylornithine |  | Yes | novel |
| 216.1222 | N-a-Acetyl-L-arginine | HMDB04620 |  |  |
| 217.1314 | Propionylcarnitine | HMDB00824 |  |  |
| 219.0565 | N-acetyl alliin* |  | Yes | novel |
| 219.1107 | Pantothenic acid | HMDB00210 | Yes |  |
| 221.0899 | N-Acetyl-D-glucosamine | HMDB00215 |  |  |
| 224.0797 | L-3-Hydroxykynurenine | HMDB11631 |  |  |
| 228.1110 | Pyroglutamylvaline |  | Yes | novel |
| 228.1110 | Prolylhydroxyproline | HMDB06695 | Yes |  |
| 231.1471 | Isobutyryl-L-carnitine | HMDB00736 | Yes | novel |
| 232.1212 | Melatonin | HMDB01389 |  |  |
| 237.1365 | Phenylcarnitine* |  | Yes | novel |
| 240.0238 | L-Cystine | HMDB00192 |  |  |
| 240.0998 | 3-Carboxy-4-methyl-5-propyl-2-furanpropionic acid | HMDB61112 |  |  |
| 242.0903 | Thymidine | HMDB00273 |  |  |
| 243.0855 | Cytidine | HMDB00089 | Yes |  |
| 243.1471 | Tiglylcarnitine | HMDB02366 |  |  |
| 244.0695 | Pseudouridine | HMDB00767 | Yes |  |
| 244.0695 | Uridine | HMDB00296 |  |  |
| 245.1627 | 2-Methylbutyroylcarnitine | HMDB00378 |  |  |
| 245.1627 | Isovalerylcarnitine | HMDB00688 |  |  |
| 246.0198 | 3-[3-(sulfooxy)phenyl]propanoic acid | | Yes | novel |
| 246.1004 | N-acetyltryptophan | HMDB13713 | Yes |  |
| 247.0151 | 2-Hydroxyacetaminophen sulfate* | | Yes | novel |
| 248.1412 | Gamma-CEHC | HMDB01931 | Yes |  |
| 251.1018 | Deoxyadenosine | HMDB00101 |  |  |
| 253.0811 | Neopterin | HMDB00845 |  |  |
| 260.1372 | L-gamma-glutamyl-L-isoleucine | HMDB11170 | Yes | novel |
| 261.0307 | O-sulfo-L-tyrosine |  | Yes | novel |
| 262.0147 | Homovanillic acid sulfate | HMDB11719 | Yes | novel |
| 264.0304 | 3-Methoxy-4-hydroxyphenylethyleneglycol sulfate | HMDB00559 |  |  |
| 264.1110 | Alpha-N-Phenylacetyl-L-glutamine | HMDB06344 | Yes |  |
| 268.0551 | DL-Homocystine | HMDB00575 |  |  |
| 268.1172 | Acetylcarnosine | HMDB12881 | Yes | novel |
| 270.0740 | Phenol glucuronide | HMDB60014 |  |  |
| 270.0852 | 4-Pyridone-3-carboxamide-1-β-d-ribonucleoside |  |  |  |
| 275.1369 | Glutarylcarnitine | HMDB13130 | Yes |  |
| 281.1124 | 1-Methyladenosine | HMDB03331 |  |  |
| 281.1124 | N6-Methyladenosine | HMDB04044 |  |  |
| 282.0964 | 1-Methylinosine | HMDB02721 |  |  |
| 283.0917 | 8-Hydroxy-deoxyguanosine | HMDB03333 |  |  |
| 284.0757 | Xanthosine | HMDB00299 |  |  |
| 284.0896 | p-Cresol glucuronide | HMDB11686 |  |  |
| 285.0961 | N4-Acetylcytidine | HMDB05923 |  |  |
| 287.2097 | L-Octanoylcarnitine | HMDB00791 |  |  |
| 288.0594 | Orotidine | HMDB00788 |  |  |
| 289.1274 | Ophthalmic acid | HMDB05765 |  |  |
| 289.1525 | 3-Methylglutarylcarnitine | HMDB00552 | Yes |  |
| 297.0896 | 5'-Methylthioadenosine | HMDB01173 | Yes | novel |
| 297.1073 | 1-Methylguanosine | HMDB01563 |  |  |
| 303.1219 | Indoleacetyl glutamine | HMDB13240 | Yes | novel |
| 308.1584 | 1-[(5-Amino-5-carboxypentyl)amino]-1-deoxyfructose | HMDB34879 |  |  |
| 309.0849 | Indoxyl glucuronide | HMDB10319 |  |  |
| 309.1060 | N-Acetylneuraminic acid | HMDB00230 | Yes |  |
| 311.1230 | N2,N2-Dimethylguanosine | HMDB04824 | Yes |  |
| 315.2410 | Decanoylcarnitine | HMDB00651 |  |  |
| 342.1162 | Alpha-Lactose | HMDB00186 |  |  |
| 342.1162 | Sucrose | HMDB00258 | Yes |  |
| 345.0474 | Cyclic GMP | HMDB01314 |  |  |
| 366.1427 | C-mannosyltryptophan |  | Yes |  |
| 370.1814 | Androsterone sulfate | HMDB02759 | Yes |  |
| 371.0852 | Salicyluric glucuronide* |  | Yes |  |
| 376.1383 | Riboflavin | HMDB00244 | Yes |  |
| 378.2016 | Pentosidine | HMDB03933 |  |  |
| 384.1216 | S-Adenosylhomocysteine | HMDB00939 | Yes |  |
| 412.1343 | N6-Carbamoyl-L-threonyladenosine | HMDB41623 | Yes |  |
| 424.1733 | Gamma-CEHC glucuronide* |  | Yes | novel |
| 454.1839 | Alpha-CEHC glucuronide* |  | Yes | novel |
| 492.1488 | 21-Hydroxypregnenolone disulfate |  |  |  |
| 573.2257 | Methionine-enkephalin |  |  |  |
| 674.2382 | 6-Sialyl-N-acetyllactosamine | HMDB06584 | Yes | novel |

There are 48 compounds marked "novel" in the reference column because they were identified as uremic in the present study we could not find prior references to their being uremic solutes. HD/Nl concentration ratios were calculated only when measureable peak areas were obtained in at least two control samples. * indicates a solute for which a reagent standard was not run but for which identity was considered well established by MS/MS. ^§^ indicates that the analysis does not distinguish between the D- and L- forms of arabitol. ^†^ indicates that the analytic method did not distinguish which OH group on 3-Methylcatehol had been sulfated.

**References to S4 Table**

1. Duranton F, Cohen G, De Smet R, Rodriguez M, Jankowski J, Vanholder R, et al. Normal and pathologic concentrations of uremic toxins. J Am Soc Nephrol. 2012;23(7):1258-70.

2. Vanholder R, De Smet R, Glorieux G, Argiles A, Baurmeister U, Brunet P, et al. Review on uremic toxins: classification, concentration, and interindividual variability. Kidney Int. 2003;63(5):1934-43.

3. Niwa T. Update of uremic toxin research by mass spectrometry. Mass Spectrom Rev. 2011;30(3):510-21.

4. Flugel-Link RM, Jones MR, Kopple JD. Red cell and plasma amino acid concentrations in renal failure. JPEN J Parenter Enteral Nutr. 1983;7(5):450-6.

5. Chuang CK, Lin SP, Chen HH, Chen YC, Wang TJ, Shieh WH, et al. Plasma free amino acids and their metabolites in Taiwanese patients on hemodialysis and continuous ambulatory peritoneal dialysis. Clin Chim Acta. 2006;364(1-2):209-16.

6. Rhee EP, Souza A, Farrell L, Pollak MR, Lewis GD, Steele DJ, et al. Metabolite profiling identifies markers of uremia. J Am Soc Nephrol. 2010;21(6):1041-51.

7. Toyohara T, Akiyama Y, Suzuki T, Takeuchi Y, Mishima E, Tanemoto M, et al. Metabolomic profiling of uremic solutes in CKD patients. Hypertens Res. 2010;33(9):944-52.

8. Gejyo F, Kinoshita Y, Ikenaka T. Identification of beta-aminoisobutyric acid in uremic serum. Clin Chim Acta. 1976;70(3):407-15.

9. Gejyo F, Kinoshita Y, Ikenaka T. Elevation of serum levels of beta-aminoisobutyric acid in uremic patients and the toxicity of the amino acid. Clin Nephrol. 1977;8(6):520-5.

10. Yu B, Zheng Y, Nettleton JA, Alexander D, Coresh J, Boerwinkle E. Serum metabolomic profiling and incident CKD among African Americans. Clin J Am Soc Nephrol. 2014;9(8):1410-7.

11. Niwa T, Maeda K, Ohki T, Saito A, Kobayashi K. A gas chromatographic-mass spectrometric analysis for phenols in uremic serum. Clin Chim Acta. 1981;110(1):51-7.

12. Sato E, Kohno M, Yamamoto M, Fujisawa T, Fujiwara K, Tanaka N. Metabolomic analysis of human plasma from haemodialysis patients. Eur J Clin Invest. 2011;41(3):241-55.

13. Itoh Y, Ezawa A, Kikuchi K, Tsuruta Y, Niwa T. Protein-bound uremic toxins in hemodialysis patients measured by liquid chromatography/tandem mass spectrometry and their effects on endothelial ROS production. Anal Bioanal Chem. 2012;403(7):1841-50.

14. Biasioli S, Feriani M, Bigi L, Dell'Aquila R, Bragantini L, Chiaramonte S, et al. Tricarboxylic acid cycle intermediates in chronic renal failure. Nephrol Dial Transplant. 1987;2(5):313-5.

15. Sirich TL, Aronov PA, Plummer NS, Hostetter TH, Meyer TW. Numerous protein-bound solutes are cleared by the kidney with high efficiency. Kidney Int. 2013;84(3):585-90.

16. Aronov PA, Luo FJ, Plummer NS, Quan Z, Holmes S, Hostetter TH, et al. Colonic contribution to uremic solutes. J Am Soc Nephrol. 2011;22(9):1769-76.

17. Bultitude FW, Newham SJ. Identification of some abnormal metabolites in plasma from uremic subjects. Clin Chem. 1975;21(9):1329-34.

18. Niwa T, Ohki T, Maeda K, Saito A, Ohta K, Kobayashi K. A gas chromatographic-mass spectrometric assay for nine hydroxyphenolic acids in uremic serum. Clin Chim Acta. 1979;96(3):247-54.

19. Boelaert J, d'kindt R, Schepers E, Jorge L, Glorieux G, Neirynck N, et al. State-of-the-are non-targeted metabolomics in the study of chronic kidney disease. Metabolomics. 2014;10:425-42.

20. Laidlaw SA, Berg RL, Kopple JD, Naito H, Walker WG, Walser M. Patterns of fasting plasma amino acid levels in chronic renal insufficiency: results from the feasibility phase of the Modification of Diet in Renal Disease Study. Am J Kidney Dis. 1994;23(4):504-13.

21. Russell RG, Bisaz S, Fleisch H. Pyrophosphate and diphosphonates in calcium metabolism and their possible role in renal failure. Arch Intern Med. 1969;124(5):571-7.

22. Silcox DC, McCarty DJ. Measurement of inorganic pyrophosphate in biological fluids. Elevated levels in some patients with osteoarthritis, pseudogout, acromegaly, and uremia. J Clin Invest. 1973;52(8):1863-70.

23. Saito A, Niwa T, Maeda K, Kobayashi K, Yamamoto Y, Ohta K. Tryptophan and indolic tryptophan metabolites in chronic renal failure. Am J Clin Nutr. 1980;33(7):1402-6.

24. Niwa T, Yamamoto N, Maeda K, Yamada K, Ohki T, Mori M. Gas chromatographic--mass spectrometric analysis of polyols in urine and serum of uremic patients. Identification of new deoxyalditols and inositol isomers. J Chromatogr. 1983;277:25-39.

25. Koeth RA, Kalantar-Zadeh K, Wang Z, Fu X, Tang WH, Hazen SL. Protein carbamylation predicts mortality in ESRD. J Am Soc Nephrol. 2013;24(5):853-61.

26. Suh B, Lee HW, Hong SY, Kim S, Eshraghi J, Paik WK. A new HPLC analytical method for o-hydroxyhippuric acid in uremic serum. J Biochem Biophys Methods. 1986;13(4-5):211-20.

27. Pawlak D, Pawlak K, Malyszko J, Mysliwiec M, Buczko W. Accumulation of toxic products degradation of kynurenine in hemodialyzed patients. Int Urol Nephrol. 2001;33(2):399-404.

28. Wlodek PJ, Iciek MB, Milkowski A, Smolenski OB. Various forms of plasma cysteine and its metabolites in patients undergoing hemodialysis. Clin Chim Acta. 2001;304(1-2):9-18.

29. Byrd DJ, Berthold HW, Trefz KF, Kochen W, Gilli G, Scharer K, et al. Indolic tryptophan metabolism in uraemia. Proc Eur Dial Transplant Assoc. 1976;12:347-54.

30. Himmelfarb J, Kane J, McMonagle E, Zaltas E, Bobzin S, Boddupalli S, et al. Alpha and gamma tocopherol metabolism in healthy subjects and patients with end-stage renal disease. Kidney Int. 2003;64(3):978-91.

31. Galli F, Floridi AG, Floridi A, Buoncristiani U. Accumulation of vitamin E metabolites in the blood of renal failure patients. Clin Nutr. 2004;23(2):205-12.

32. Ozben T. Elevated serum and urine sialic acid levels in renal diseases. Ann Clin Biochem. 1991;28 ( Pt 1):44-8.

33. Tseke P, Grapsa E, Stamatelopoulos K, Samouilidou E, Rammos G, Papamichael C, et al. Correlations of sialic acid with markers of inflammation, atherosclerosis and cardiovascular events in hemodialysis patients. Blood Purif. 2008;26(3):261-6.

34. Niewczas MA, Sirich TL, Mathew AV, Skupien J, Mohney RP, Warram JH, et al. Uremic solutes and risk of end-stage renal disease in type 2 diabetes: metabolomic study. Kidney Int. 2014;85(5):1214-24.

35. Szucs J, Bodrogi L. Androsterone sulphate, dehydroepiandrosterone sulphate and free dehydroepiandrosterone in uraemic patients. Horm Metab Res. 1980;12(8):417-8.

36. Takeshi I, Niwa T, Matsui E. Vitamin B2 and Vitamin E in long-term hemodialysis. JAMA. 1971;217(5):699.

37. Kamata K, Okubo M, Marumo F. Water soluble vitamins in patients with chronic renal failure and effect of B6 administration of immunological activity. Proc Clin Dial Transplant Forum. 1979;9:194-6.

38. DeBari VA, Frank O, Baker H, Needle MA. Water soluble vitamins in granulocytes, erythrocytes, and plasma obtained from chronic hemodialysis patients. Am J Clin Nutr. 1984;39(3):410-5.

39. Marumo F, Kamata K, Okubo M. Deranged concentrations of water-soluble vitamins in the blood of undialyzed and dialyzed patients with chronic renal failure. Int J Artif Organs. 1986;9(1):17-24.
