# Supplementary material for: An Enlarged Profile of Uremic Solutes
Source: PLoS One. 2015 Aug 28;10(8):e0135657. doi: 10.1371/journal.pone.0135657 (PMC4552739; doi:10.1371/journal.pone.0135657)
Supplement: S5 Table — (DOCX) [file pone.0135657.s005.docx]

**S5 Table. Solutes Detected in Plasma and Categorized as Uremic in Some Previous Studies but not in the Present Study (n=28)**

| Solute | HMDB Citation | Mass Da | #HD/#Nl Total Plasma | #HD/#Nl Plasma  Ultrafiltrate | HD/Nl Total Plasma | HD/Nl Plasma Ultrafiltrate |
| --- | --- | --- | --- | --- | --- | --- |
| Dimethylglycine | HMDB00092 | 103 | 6/6 | 6/6 | 2.2 | 2.3 |
| Choline | HMDB00097 | 104 | 6/6 | 6/6 | 1.8 | 1.8 |
| Uracil | HMDB00300 | 112 | 5/6 | 6/6 | 1.2 | 1.3 |
| Succinic acid | HMDB00254 | 118 | 6/6 | 6/6 | 1.7 | 1.4 |
| 4-Hydroxyproline | HMDB00725 | 131 | 6/6 | 5/5 | 2.3 | 2.4 |
| Creatine | HMDB00064 | 131 | 6/6 | 6/6 | 0.6 | 0.4 |
| Hypoxanthine | HMDB00157 | 136 | 6/6 | 6/6 | 1.0 | 0.6 |
| Salicylic acid | HMDB01895 | 138 | 2/1 | 2/1 | 1.0 | 2.7 |
| 4-Trimethylammoniobutanoic acid | HMDB01161 | 146 | 6/6 | 5/6 | 1.4 | 1.0 |
| Xanthine | HMDB00292 | 152 | 6/6 | 6/6 | 0.9 | 1.1 |
| 7-Methylguanine | HMDB00897 | 165 | 6/6 | 6/6 | 1.6 | 1.8 |
| Uric acid | HMDB00289 | 168 | 6/6 | 6/6 | 0.7 | 1.3 |
| Suberic acid | HMDB00893 | 174 | 6/6 | 6/6 | 0.8 | 0.8 |
| Pyrophosphate | HMDB00250 | 174 | 3/3 | 5/3 |  |  |
| Sorbitol | HMDB00247 | 182 | 2/6 | 2/6 | 2.4 | 3.3 |
| Azelaic acid | HMDB00784 | 188 | 6/6 | 6/6 | 0.5 | 0.5 |
| Citric acid | HMDB00094 | 192 | 6/6 | 6/6 | 1.7 | 1.8 |
| Quinic acid | HMDB03072 | 192 | 6/5 | 6/5 | 2.2 | 1.6 |
| Sebacic acid | HMDB00792 | 202 | 6/6 | 6/6 | 0.4 | 0.6 |
| Asymmetric dimethylarginine^†^ /  Symmetric dimethylarginine^†^ | HMDB01539 HMDB03334 | 202 | 6/5 | 6/6 | 1.3 | 1.6 |
| L-Kynurenine | HMDB00684 | 208 | 6/6 | 6/6 | 1.7 | 2.3 |
| 3-Carboxy-4-methyl-5-propyl-2-furanpropionic acid | HMDB61112 | 240 | 6/6 | 0/0 | 1.3 |  |
| Uridine | HMDB00296 | 244 | 6/6 | 6/6 | 0.3 | 0.3 |
| 1-Methyladenosine | HMDB03331 | 281 | 6/6 | 6/6 | 1.7 | 1.2 |
| N6-Methyladenosine | HMDB04044 | 281 | 6/6 | 6/6 | 1.5 | 1.2 |
| Xanthosine | HMDB00299 | 284 | 1/1 | 2/1 | 86.3 | 43.4 |
| 1-Methylguanosine | HMDB01563 | 297 | 6/3 | 6/6 | 1.8 | 2.3 |

This table lists those 28 solutes among the 459 solutes detected when comparing plasma samples from dialysis patients and controls in the present study which had previously reported as uremic but were not classified as uremic by the criteria employed in the current study. #HD/#Nl represents the numbers that we detected the solute in plasma or plasma ultrafiltrate of HD patients or normal subjects. HD/Nl represents concentration ratios predialysis compared to normal in plasma or plasma ultrafiltrate, as estimated from mass spectrometric peak areas. ^†^ indicates that the analytic method did not distinguish between asymmetric and symmetric dimethylarginine. References list previous reports of the solutes' accumulation in renal failure.
