# Supplementary material for: An Enlarged Profile of Uremic Solutes
Source: PLoS One. 2015 Aug 28;10(8):e0135657. doi: 10.1371/journal.pone.0135657 (PMC4552739; doi:10.1371/journal.pone.0135657)
Supplement: S6 Table — (DOC) [file pone.0135657.s006.doc]

**S6 Table. Plant Sources of Uremic Phenyl and Indole Solutes**

Literature search revealed plant sources for most of the uremic phenyl and indole compounds which are not produced by amino acid degradation in mammalian cells, as listed in Table 2 of the main report and again listed in the first column here. Studies cited in the second column suggest that the unconjugated form of the uremic solute is itself found in plant foods. Those in the third column describe derivation of these compounds from metabolism of plant food compounds, frequently identified as polyphenols and/or phenolic acids. Metabolism of these plant compounds is often accomplished by colon microbes. Studies reporting production of specific solutes by colon microbes are cited in the fourth column. Studies reporting other sources of the compounds are cited in the fifth column. Exceptions to the common pattern of derivation from plant foods are homovanillic acid sulfate and vanillylmandelic acid which are mammalian degradation products of dopamine and epinephrine/norepinephrine. P-cresol sulfate and indoxyl sulfate are derived from microbial metabolism of the amino acids phenylalanine/tyrosine and tryptophan which may reach the colon from either animal foods, plant foods, or internal secretions. We could not find reference to the source of phenol, but suspect it is derived from plant phenols and polyphenols.

| Solute | Found in Plant Foods | Derived From Plant Compounds | Microbial Metabolism of Plant Compounds | Other Sources |
| --- | --- | --- | --- | --- |
| Phenylacetic acid |  | Polyphenols  and  Phenols |  | Microbial metabolism of phenylalanine |
| 2-Aminobenzoic acid | Corn |  |  | Mammalian metabolism of tryptophan |
| 4-Hydroxyphenylacetic acid | Plum  Beer | Polyphenols  and  Phenols |  |  |
| Vanillic acid | Plum  Beer  Strawberry  Cauliflower  Barley | Polyphenols  and  Phenols |  |  |
| Phenol sulphate |  |  |  |  |
| Hippuric acid | Mushroom  Saffron corms  Sage tea | Polyphenols  and  Phenols |  |  |
| p-Cresol sulfate |  |  |  | Mammalian metabolism of phenylalanine and tyrosine |
| 2-Aminophenol sulphate |  | Whole grain rye bread  Plant dietary fiber |  |  |
| Pyrocatechol sulfate | Coffee,  Beer,  Malt,  Bread crust, Cocoa,  Potato crisps, Peach,  Apple,  Mango  Coffee | Polyphenols  and  Phenols |  |  |
| Phenylacetylglycine *(glycine conjugate of phenylacetate above)* |  |  |  |  |
| 4-Hydroxyhippuric acid |  | Polyphenols |  |  |
| 3-Hydroxyhippuric acid |  | Polyphenols |  |  |

| Vanillylmandelic acid |  | Bananas |  | Mammalian catabolism of catecholamines |
| --- | --- | --- | --- | --- |
| 4-Vinylphenol sulfate | Beer  Cherry  Wine  Apple Cider |  |  |  |
| 2-Methoxyphenol sulphate | Coffee  Sesame oil |  |  |  |
| 3-Methylcatechol sulfate 1 | Coffee,  Dark beer, Malt,  Bread crust, Cocoa | Polyphenols  and  Phenols |  |  |
| 4-Methylcatechol sulfate | Coffee,  Dark beer, Malt,  Bread crust, Cocoa | Polyphenols  and  Phenols |  |  |
| Cinnamoylglycine | Cranberry  Mangosteen fruit  Mushroom  Apple  Grape | Polyphenols |  |  |
| 3-[3-(Sulfooxy)phenyl]propanoic acid |  | Polyphenols  and  Phenols |  |  |

| Homovanillic acid sulfate | Olive oil |  |  | Mammalian catabolism of dopamine |
| --- | --- | --- | --- | --- |
| Alpha-N-Phenylacetyl-L-glutamine *(glutamine conjugate of phenylacetate above)* |  |  |  |  |
| Indole-3-methyl acetate | Apple |  |  |  |
| 2-oxindole-3-acetate | Corn-Gluten meal |  |  |  |
| Indoxyl sulfate |  |  |  | Microbial metabolism of tryptophan |

**References to S6 Table**

1. Williamson G, Clifford MN. Colonic metabolites of berry polyphenols: the missing link to biological activity? Br J Nutr. 2010;104 Suppl 3:S48-66.

2. Gill CI, McDougall GJ, Glidewell S, Stewart D, Shen Q, Tuohy K, et al. Profiling of phenols in human fecal water after raspberry supplementation. J Agric Food Chem. 2010;58(19):10389-95.

3. Urpi-Sarda M, Monagas M, Khan N, Llorach R, Lamuela-Raventos RM, Jauregui O, et al. Targeted metabolic profiling of phenolics in urine and plasma after regular consumption of cocoa by liquid chromatography-tandem mass spectrometry. J Chromatogr A. 2009;1216(43):7258-67.

4. Nieman DC, Gillitt ND, Knab AM, Shanely RA, Pappan KL, Jin F, et al. Influence of a polyphenol-enriched protein powder on exercise-induced inflammation and oxidative stress in athletes: a randomized trial using a metabolomics approach. PLoS One. 2013;8(8):e72215.

5. Clayton TA, Baker D, Lindon JC, Everett JR, Nicholson JK. Pharmacometabonomic identification of a significant host-microbiome metabolic interaction affecting human drug metabolism. Proc Natl Acad Sci U S A. 2009;106(34):14728-33.

6. Smith EA, Macfarlane GT. Formation of Phenolic and Indolic Compounds by Anaerobic Bacteria in the Human Large Intestine. Microb Ecol. 1997;33(3):180-8.

7. Singh M, Widholm JM. Study of a corn (Zea mays L.) mutant (blue fluorescent-1) which accumulates anthranilic acid and its beta-glucoside. Biochem Genet. 1975;13(5-6):357-67.

8. Kolodziej LR, Paleolog EM, Williams RO. Kynurenine metabolism in health and disease. Amino Acids. 2011;41(5):1173-83.

9. Sallee M, Dou L, Cerini C, Poitevin S, Brunet P, Burtey S. The aryl hydrocarbon receptor-activating effect of uremic toxins from tryptophan metabolism: a new concept to understand cardiovascular complications of chronic kidney disease. Toxins (Basel). 2014;6(3):934-49.

10. Vanholder R, Schepers E, Pletinck A, Nagler EV, Glorieux G. The uremic toxicity of indoxyl sulfate and p-cresyl sulfate: a systematic review. J Am Soc Nephrol. 2014;25(9):1897-907.

11. Biesaga M, Ochnik U, Pyrzynska K. Analysis of phenolic acids in fruits by HPLC with monolithic columns. J Sep Sci. 2007;30(17):2929-34.

12. Nardini M, Natella F, Scaccini C, Ghiselli A. Phenolic acids from beer are absorbed and extensively metabolized in humans. J Nutr Biochem. 2006;17(1):14-22.

13. Roowi S, Stalmach A, Mullen W, Lean ME, Edwards CA, Crozier A. Green tea flavan-3-ols: colonic degradation and urinary excretion of catabolites by humans. J Agric Food Chem. 2010;58(2):1296-304.

14. McKay DL, Chen CY, Zampariello CA, Blumberg JB. Flavonoids and phenolic acids from cranberry juice are bioavailable and bioactive in healthy older adults. Food Chem. 2015;168:233-40.

15. Russell WR, Scobbie L, Labat A, Duthie GG. Selective bio-availability of phenolic acids from Scottish strawberries. Mol Nutr Food Res. 2009;53 Suppl 1:S85-91.

16. Ahmed FA, Ali RF. Bioactive compounds and antioxidant activity of fresh and processed white cauliflower. Biomed Res Int. 2013;2013:367819.

17. Hao M, Beta T. Qualitative and quantitative analysis of the major phenolic compounds as antioxidants in barley and flaxseed hulls using HPLC/MS/MS. J Sci Food Agric. 2012;92(10):2062-8.

18. Nurmi T, Mursu J, Heinonen M, Nurmi A, Hiltunen R, Voutilainen S. Metabolism of berry anthocyanins to phenolic acids in humans. J Agric Food Chem. 2009;57(6):2274-81.

19. Rios LY, Gonthier MP, Remesy C, Mila I, Lapierre C, Lazarus SA, et al. Chocolate intake increases urinary excretion of polyphenol-derived phenolic acids in healthy human subjects. Am J Clin Nutr. 2003;77(4):912-8.

20. Rechner AR, Kuhnle G, Hu H, Roedig-Penman A, van den Braak MH, Moore KP, et al. The metabolism of dietary polyphenols and the relevance to circulating levels of conjugated metabolites. Free Radic Res. 2002;36(11):1229-41.

21. Khoddami A, Wilkes MA, Roberts TH. Techniques for analysis of plant phenolic compounds. Molecules. 2013;18(2):2328-75.

22. Olthof MR, Hollman PC, Buijsman MN, van Amelsvoort JM, Katan MB. Chlorogenic acid, quercetin-3-rutinoside and black tea phenols are extensively metabolized in humans. J Nutr. 2003;133(6):1806-14.

23. Bondia-Pons I, Barri T, Hanhineva K, Juntunen K, Dragsted LO, Mykkanen H, et al. UPLC-QTOF/MS metabolic profiling unveils urinary changes in humans after a whole grain rye versus refined wheat bread intervention. Mol Nutr Food Res. 2013;57(3):412-22.

24. Johansson-Persson A, Barri T, Ulmius M, Onning G, Dragsted LO. LC-QTOF/MS metabolomic profiles in human plasma after a 5-week high dietary fiber intake. Anal Bioanal Chem. 2013;405(14):4799-809.

25. Lang R, Mueller C, Hofmann T. Development of a stable isotope dilution analysis with liquid chromatography-tandem mass spectrometry detection for the quantitative analysis of di- and trihydroxybenzenes in foods and model systems. J Agric Food Chem. 2006;54(16):5755-62.

26. Fujioka K, Shibamoto T. Quantitation of volatiles and nonvolatile acids in an extract from coffee beverages: correlation with antioxidant activity. J Agric Food Chem. 2006;54(16):6054-8.

27. Jaganath IB, Mullen W, Edwards CA, Crozier A. The relative contribution of the small and large intestine to the absorption and metabolism of rutin in man. Free Radic Res. 2006;40(10):1035-46.

28. Odink J, Korthals H, Knijff JH. Simultaneous determination of the major acidic metabolites of catecholamines and serotonin in urine by liquid chromatography with electrochemical detection after a one-step sample clean-up on Sephadex G-10; influence of vanilla and banana ingestion. J Chromatogr. 1988;424(2):273-83.

29. Eisenhofer G, Kopin IJ, Goldstein DS. Catecholamine metabolism: a contemporary view with implications for physiology and medicine. Pharmacol Rev. 2004;56(3):331-49.

30. Vanbeneden N, Delvaux F, Delvaux FR. Determination of hydroxycinnamic acids and volatile phenols in wort and beer by isocratic high-performance liquid chromatography using electrochemical detection. J Chromatogr A. 2006;1136(2):237-42.

31. Wen YQ, He F, Zhu BQ, Lan YB, Pan QH, Li CY, et al. Free and glycosidically bound aroma compounds in cherry (Prunus avium L.). Food Chem. 2014;152:29-36.

32. Silva I, Campos FM, Hogg T, Couto JA. Factors influencing the production of volatile phenols by wine lactic acid bacteria. Int J Food Microbiol. 2011;145(2-3):471-5.

33. Xu Y, Fan W, Qian MC. Characterization of aroma compounds in apple cider using solvent-assisted flavor evaporation and headspace solid-phase microextraction. J Agric Food Chem. 2007;55(8):3051-7.

34. Shimoda M NY, Nakashima M, Osajima Y. Quantitative Comparison of Volatile Flavor Compounds in Deep-Roasted and Light-Roasted Sesame Seed Oil. J Agric Food Chem. 1997;45(8):3193-6.

35. Ye M, Yue T, Yuan Y. Evolution of polyphenols and organic acids during the fermentation of apple cider. J Sci Food Agric. 2014;94(14):2951-7.

36. Nile SH, Kim SH, Ko EY, Park SW. Polyphenolic contents and antioxidant properties of different grape (V. vinifera, V. labrusca, and V. hybrid) cultivars. Biomed Res Int. 2013;2013:718065.

37. Konishi Y, Kobayashi S. Microbial metabolites of ingested caffeic acid are absorbed by the monocarboxylic acid transporter (MCT) in intestinal Caco-2 cell monolayers. J Agric Food Chem. 2004;52(21):6418-24.

38. Tuck KL, Hayball PJ. Major phenolic compounds in olive oil: metabolism and health effects. J Nutr Biochem. 2002;13(11):636-44.

39. Orozco-Solano MI, Ferreiro-Vera C, Priego-Capote F, Luque de Castro MD. Automated method for determination of olive oil phenols and metabolites in human plasma and application in intervention studies. J Chromatogr A. 2012;1258:108-16.

40. Vine JH, Noiton D, Plummer JA, Baleriola-Lucas C, Mullins MG. Simultaneous quantitation of indole 3-acetic Acid and abscisic Acid in small samples of plant tissue by gas chromatography/mass spectrometry/selected ion monitoring. Plant Physiol. 1987;85(2):419-22.

41. Niwa T, Ishii S, Hiramatsu A, Osawa T. Oxidative reaction of oxindole-3-acetic acids. Biosci Biotechnol Biochem. 2003;67(9):1870-4.
